# Comparative secretome analysis of *Streptomyces scabiei* during growth in the presence or absence of potato suberin

**DOI:** 10.1186/1477-5956-12-35

**Published:** 2014-06-25

**Authors:** Doaa Komeil, Rebeca Padilla-Reynaud, Sylvain Lerat, Anne-Marie Simao-Beaunoir, Carole Beaulieu

**Affiliations:** 1 Centre SÈVE, Département de biologie, Université de Sherbrooke, Québec J1K 2R1, Canada; 2 Department of Plant Pathology, Faculty of Agriculture, University of Alexandria, El-Shatby 21545, Egypt

**Keywords:** *Streptomyces scabies*, Common scab, Proteomics, Feruloyl esterase, Glycosyl hydrolase, Lipid metabolism, Suberinase

## Abstract

**Background:**

Suberin is a recalcitrant plant biopolymer composed of a polyphenolic and a polyaliphatic domain. Although suberin contributes to a significant portion of soil organic matter, the biological process of suberin degradation is poorly characterized. It has been suggested that *Streptomyces scabiei*, a plant pathogenic bacterium, can produce suberin-degrading enzymes. In this study, a comparative analysis of the *S. scabiei* secretome from culture media supplemented or not with potato suberin was carried out to identify enzymes that could be involved in suberin degradation.

**Methods:**

*S. scabiei* was grown in the presence of casein only or in the presence of both casein and suberin. Extracellular proteins from 1-, 3- and 5-day-old supernatants were analyzed by LC-MS/MS to determine their putative functions. Real-time RT-PCR was performed to monitor the expression level of genes encoding several proteins potentially involved in suberin degradation.

**Results:**

The effect of suberin on the extracellular protein profile of *S. scabiei* strain has been analyzed. A total of 246 proteins were found to be common in the data sets from both casein medium (CM) and casein-suberin medium (CSM), whereas 124 and 139 proteins were detected only in CM or CSM, respectively. The identified proteins could be divided into 19 functional groups. Two functional groups of proteins (degradation of aromatic compounds and secondary metabolism) were only associated with the CSM. A high proportion of the proteins found to be either exclusively produced, or overproduced, in presence of suberin were involved in carbohydrate metabolism. Most of the proteins included in the lipid metabolism class have been detected in CSM. Apart from lipid metabolism proteins, other identified proteins, particularly two feruloyl esterases, may also actively participate in the breakdown of suberin architecture. Both feruloyl esterase genes were overexpressed between 30 to 340 times in the presence of suberin.

**Conclusion:**

This study demonstrated that the presence of suberin in *S. scabiei* growth medium induced the production of a wide variety of glycosyl hydrolases. Furthermore, this study has allowed the identification of extracellular enzymes that could be involved in the degradation of suberin, including enzymes of the lipid metabolism and feruloyl esterases.

## Background

Proteomics has been successfully applied to analyze both intracellular proteins and the secretomes of several microorganisms, including plant pathogens [1]. Both the intracellular proteome [2] and the secretome [3] of the plant pathogenic bacterium *Streptomyces scabiei* have been analyzed. This pathogen is the predominant causal agent of potato common scab and causes important economic losses in most potato growing-areas [4]. The disease is characterized by shallow, raised, or deep-pitted corky-like lesions on the tuber surface. *S. scabiei* produces toxins called thaxtomins, which cause hypertrophy and cell death in host plant tissues, and are essential for pathogenicity [4].

Thaxtomin biosynthetic genes are expressed during secondary metabolism in the presence of compounds associated with tuber cell walls: cellobiose and suberin [5]. The intracellular proteomes of *S. scabiei* grown with or without suberin have previously been compared [2]. The addition of the plant polymer to the growth media resulted in an increase in proteins involved in stress response, glycolysis and morphological differentiation. Suberin also appeared to affect secondary metabolism as it caused the overproduction of BldK proteins, which are known to be involved in differentiation and secondary metabolism [2]. Suberin is also known to promote differentiation and secondary metabolism in different *Streptomyces* species [6].

Suberin is a major constituent of potato skin. This polymer is composed of two spatially distinct but covalently-linked domains; the polyphenolic domain embedded in the primary cell wall, and the polyaliphatic domain [7]. Suberized lamellae are located between the primary cell wall and the plasma membrane [7]. The polyaromatic domain is a lignin-like structure that mostly contains polyhydroxycinnamates such as feruloyltyramine [7]. The aliphatic moiety of suberin is mainly composed of ω-hydroxyacids, α,ω-diacids, fatty acids, primary alcohols and glycerol [8,9]. Glycerol may account for up to 25% of the total suberin monomers [10]. Nevertheless, the molecular structure of suberin remains speculative although the most recent models propose that ferulic acids link the aliphatic polyester domain of suberin to the neighboring polyaromatics [9,10].

Suberin is one of the most recalcitrant plant molecular structures in nature [6] and microbial degradation of suberin is a process that is poorly characterized. Suberinases are polyesterases produced by a number of fungi that can at least partially depolymerize the lipidic polymer [11]. Some authors have suggested that *S. scabiei* can also produce suberin-degrading esterases [12] that may be involved in pathogenicity. The purpose of this study was to identify enzymes that could potentially be involved in suberin degradation. *S. scabiei* EF-35 was grown in culture media containing casein as the sole carbon source or in media containing both casein and suberin. The secretomes associated with these growth conditions were then compared. Enzymes involved in both polysaccharide catabolism and lipid metabolism were up-regulated in the presence of suberin.

## Results and discussion

### Comparative analysis of the *S. scabiei* EF-35 secretome in the presence or absence of suberin

A previous study has allowed the identification, in *S. scabiei* EF-35, of intracellular soluble proteins that were differentially produced in the presence of suberin [2]. Furthermore, the twin arginine protein transport pathway secretome of another *S. scabiei* strain has been characterized by 2-D electrophoresis in four different culture media (instant potato mash medium, soy-flour mannitol medium, R5 medium and oat bran medium) [3]. In the present study, the effect of suberin, a polymer associated with potato tuber periderm, on the extracellular protein profile of *S. scabiei* EF-35 has been analyzed. Extracellular protein profiles of supernatants from *S. scabiei* EF-35 cultures grown in the presence of casein only or in the presence of both casein and suberin were compared after 1, 3 and 5 days of growth. The proteins were fractionated by one-dimensional electrophoresis and analyzed by LC-MS/MS, as fractionation of secretomes has been shown to increase the overall number of identified proteins by approximately 30% [13].

A total of 907 different proteins were found in at least one of the media tested and 509 proteins met the filtering criteria. Protein abundance was estimated by spectral counting/protein molecular weight (kDa). While 263 out of 509 proteins were restricted to a specific medium, others were found in both culture media. Proteins found in both media are listed in supporting data (Additional file 1: Table S1). Proteins detected only in casein medium (CM) are presented in Additional file 2: Table S2. A list of proteins with a 5-fold greater abundance in the casein/suberin-containing medium (CSM) compared to CM is shown in Table 1. Proteins only found in CSM are listed in Table 2. All proteins listed have been divided into 19 functional groups based on their putative functions (Table 3).

**Table 1:** **Proteins produced by *Streptomyces scabiei *** **with a 5-fold greater abundance in casein-suberin medium**

**Protein assignation**^**a**^	**Gene assignation**	**Putative function**	**Abundance (spectrum count/MW**^**b**^**) in casein-suberin medium**			**Abundance (spectrum count/MW**^**b**^**) in casein medium**		
			**Day 1**	**Day 3**	**Day 5**	**Day 1**	**Day 3**	**Day 5**
Translational, ribosomal structure and biogenesis								
C9Z240	SCAB_25251^d^	Polyribonucleotide nucleotidyltransferase	0.04 ± 0.03	0.25 ± 0.10	0.22 ± 0.06	0.01 ± 0.01	nd^c^	0.01 ± 0.01
C9Z3N7	SCAB_25991^d^	Ribosome recycling factor	0.43 ± 0.13	nd	nd	0.08 ± 0.07	0.03 ± 0.05	0.10 ± 0.10
C9Z3P0	SCAB_26021^d^	30S ribosomal protein S2	0.23 ± 0.12	0.05 ± 0.02	nd	0.03 ± 0.05	nd	nd
C9Z3Q3	SCAB_26151^d^	50S ribosomal protein L19	0.67 ± 0.32	1.13 ± 0.56	0.68 ± 0.25	0.10 ± 0.18	nd	nd
C9YW46	SCAB_36621^d^	30S ribosomal protein S9	0.42 ± 0.24	0.35 ± 0.20	0.11 ± 0.06	0.12 ± 0.13	nd	nd
C9YW47	SCAB_36631^d^	50S ribosomal protein L13	0.25 ± 0.13	0.24 ± 0.10	0.10 ± 0.03	0.02 ± 0.04	nd	nd
C9YW61	SCAB_36771^d^	30S ribosomal protein S5	0.33 ± 0.06	0.38 ± 0.05	0.01 ± 0.02	0.13 ± 0.23	nd	nd
C9YW63	SCAB_36791^d^	50S ribosomal protein L6	0.18 ± 0.12	0.33 ± 0.16	0.02 ± 0.03	0.02 ± 0.03	nd	nd
C9YW64	SCAB_36801^d^	30S ribosomal protein S8	0.17 ± 0.15	0.64 ± 0.45	0.12 ± 0.21	0.02 ± 0.04	nd	nd
C9YW68	SCAB_36841^d^	50S ribosomal protein L14	0.21 ± 0.04	0.63 ± 0.47	0.54 ± 0.45	0.05 ± 0.09	nd	nd
C9YW72	SCAB_36881^d^	30S ribosomal protein S3	0.17 ± 0.09	0.09 ± 0.08	0.01 ± 0.03	0.02 ± 0.04	nd	nd
C9YW73	SCAB_36891^d^	50S ribosomal protein L22	0.44 ± 0.16	0.85 ± 0.60	0.67 ± 0.40	0.08 ± 0.13	nd	nd
C9YW77	SCAB_36931^d^	50S ribosomal protein L4	0.62 ± 0.13	1.00 ± 0.20	0.66 ± 0.06	0.10 ± 0.14	nd	nd
C9YW78	SCAB_36941^d^	50S ribosomal protein L3	0.55 ± 0.10	0.96 ± 0.26	0.56 ± 0.15	0.04 ± 0.08	nd	nd
C9YW94	SCAB_37111^d^	30S ribosomal protein S7	0.51 ± 0.18	0.23 ± 0.24	nd	0.04 ± 0.07	nd	nd
C9ZAN5	SCAB_4612 ^d^	30S ribosomal protein S18	0.30 ± 0.06	0.17 ± 0.18	0.06 ± 0.09	0.04 ± 0.06	nd	nd
C9Z656	SCAB_75031^d^	30S ribosomal protein S4	0.51 ± 0.32	0.45 ± 0.14	0.04 ± 0.04	0.01 ± 0.02	nd	nd
Transcription								
C9ZAB5	SCAB_30041^d^	Cyclic nucleotide-binding protein	0.03 ± 0.01	0.18 ± 0.11	0.17 ± 0.01	0.03 ± 0.06	nd	nd
Cell wall/membrane/envelope biogenesis								
C9YUC5	SCAB_4961	Glucuronoxylanase XynC	0.15 ± 0.09	0.30 ± 0.21	0.26 ± 0.10	nd	nd	0.04 ± 0.03
C9Z8V2	SCAB_45141	D-alanyl-D-alanine carboxypeptidase	0.04 ± 0.02	0.42 ± 0.07	0.40 ± 0.12	0.04 ± 0.05	nd	nd
Defense mechanisms and virulence								
C9Z785	SCAB_44161^d^	β-lactamase	0.43 ± 0.07	0.51 ± 0.17	0.45 ± 0.12	0.02 ± 0.04	nd	nd
C9Z160	SCAB_56441	Protease	0.39 ± 0.23	0.00 ± 0.01	nd	nd	0.21 ± 0.02	0.11 ± 0.05
Differentiation								
C9Z7Z7	SCAB_89661	Factor C morphological differentiation homolog	nd	0.14 ± 0.05	0.11 ± 0.06	nd	nd	0.01 ± 0.02
Energy production and conversion								
C9Z8E8	SCAB_28761^d^	ATP synthase subunit β	0.28 ± 0.06	0.48 ± 0.26	0.44 ± 0.15	0.07 ± 0.11	0.06 ± 0.07	0.13 ± 0.04
C9Z8F0	SCAB_28781^d^	ATP synthase subunit α	0.17 ± 0.02	0.45 ± 0.11	0.40 ± 0.20	0.05 ± 0.06	0.06 ± 0.05	0.06 ± 0.03
C9ZGW6	SCAB_34651^d^	Oxidoreductase	0.05 ± 0.08	0.33 ± 0.08	0.35 ± 0.07	0.02 ± 0.04	0.04 ± 0.05	0.05 ± 0.08
C9ZE86	SCAB_79151	Cytokinin dehydrogenase	0.14 ± 0.01	0.36 ± 0.08	0.35 ± 0.07	nd	0.08 ± 0.10	0.21 ± 0.03
Lipid metabolism								
C9Z707	SCAB_28481^d^	Acetyl-CoA C-acetyltransferase FadA	0.04 ± 0.03	0.02 ± 0.03	nd	0.01 ± 0.01	nd	nd
C9Z5Z2	SCAB_74351	Glycerophosphoryl diester phosphodiesterase	0.09 ± 0.02	0.23 ± 0.07	0.23 ± 0.04	nd	0.05 ± 0.05	0.03 ± 0.05
C9ZCR0	SCAB_78851	Sphingolipid ceramide N-deacylase	0.24 ± 0.07	0.12 ± 0.05	0.03 ± 0.03	nd	0.15 ± 0.18	0.04 ± 0.03
Carbohydrate metabolism								
C9ZBE6	SCAB_0631	α-L-fucosidase	0.03 ± 0.00	0.08 ± 0.02	0.06 ± 0.03	nd	nd	0.01 ± 0.02
C9YSR8	SCAB_3851	Fucosidase	0.18 ± 0.02	0.41 ± 0.16	0.35 ± 0.11	nd	nd	0.00 ± 0.01
C9YSS0	SCAB_3881	Arabinofuranosidase	0.13 ± 0.01	0.39 ± 0.13	0.32 ± 0.15	nd	0.18 ± 0.16	0.04 ± 0.00
C9YSS1	SCAB_3891	α-galactosidase	0.10 ± 0.06	0.22 ± 0.21	0.13 ± 0.11	nd	0.01 ± 0.01	nd
C9YVN3	SCAB_5851	Putative secreted protein	0.01 ± 0.01	0.04 ± 0.03	0.04 ± 0.04	nd	0.02 ± 0.01	nd
C9YVP9	SCAB_6021	Endo β-1.4-xylanase	0.29 ± 0.13	0.67 ± 0.20	0.65 ± 0.19	nd	0.04 ± 0.04	nd
C9Z0D5	SCAB_8871	Endoglucanase	0.03 ± 0.00	0.05 ± 0.02	0.08 ± 0.05	nd	0.02 ± 0.01	0.02 ± 0.01
C9Z1T6	SCAB_9291	Lactonase	nd	0.14 ± 0.05	0.20 ± 0.07	nd	0.02 ± 0.03	nd
C9Z507	SCAB_11431	Glycosyl hydrolase	0.39 ± 0.10	0.88 ± 0.30	0.64 ± 0.17	nd	0.42 ± 0.13	0.33 ± 0.14
C9Z878	SCAB_13491	Glycosyl hydrolase	nd	0.03 ± 0.02	0.04 ± 0.02	nd	nd	0.01 ± 0.01
C9ZD50	SCAB_16431	Cellulase	0.43 ± 0.29	1.52 ± 0.86	1.04 ± 0.61	nd	0.93 ± 0.72	0.39 ± 0.04
C9ZD59	SCAB_16521	Arabinofuranosidase	0.38 ± 0.12	1.07 ± 0.39	0.96 ± 0.36	nd	0.33 ± 0.24	0.33 ± 0.17
C9ZEP9	SCAB_17001	Cellulase	0.05 ± 0.04	1.05 ± 0.47	1.05 ± 0.38	nd	0.29 ± 0.14	0.19 ± 0.14
C9ZEQ0	SCAB_17011	Cellulase	0.15 ± 0.04	0.85 ± 0.26	0.73 ± 0.21	nd	0.02 ± 0.02	0.02 ± 0.02
C9YT14	SCAB_19051	Solute-binding lipoprotein	nd	0.12 ± 0.05	0.21 ± 0.11	nd	0.09 ± 0.08	0.02 ± 0.02
C9YT63	SCAB_19561	β-xylosidase	0.09 ± 0.10	0.23 ± 0.17	0.24 ± 0.17	nd	0.36 ± 0.18	0.20 ± 0.05
C9YUL1	SCAB_19941	Glycosyl hydrolase	0.47 ± 0.08	0.46 ± 0.29	0.33 ± 0.18	0.04 ± 0.03	0.38 ± 0.12	0.26 ± 0.12
C9YYV2	SCAB_22931	Arabinofuranosidase	0.24 ± 0.07	0.40 ± 0.20	0.35 ± 0.14	nd	0.22 ± 0.16	0.12 ± 0.03
C9Z271	SCAB_25571	Glycosyl hydrolase	nd	0.22 ± 0.17	0.17 ± 0.07	nd	0.10 ± 0.09	nd
C9YUZ2	SCAB_36371	Xylanase/cellulase	0.24 ± 0.10	0.51 ± 0.16	0.47 ± 0.13	0.02 ± 0.02	0.23 ± 0.18	0.14 ± 0.09
C9Z5L1	SCAB_42951	Glucose / Sorbosone dehydrogenase	0.87 ± 0.12	4.30 ± 2.73	4.18 ± 1.77	0.14 ± 0.14	0.91 ± 0.35	0.60 ± 0.22
C9Z737	SCAB_43661	Galactan endo-1.6-β-galactosidase	0.20 ± 0.08	0.32 ± 0.11	0.18 ± 0.08	0.01 ± 0.02	nd	nd
C9Z7A0	SCAB_44311	α-galactosidase	0.13 ± 0.07	0.15 ± 0.03	0.12 ± 0.07	nd	0.07 ± 0.05	0.06 ± 0.05
C9Z7A1	SCAB_44321	Glycosyl hydrolase	0.17 ± 0.05	0.05 ± 0.05	0.07 ± 0.04	nd	nd	0.01 ± 0.01
C9YTK2	SCAB_51081	Cellulase	0.01 ± 0.01	0.57 ± 0.25	0.46 ± 0.13	nd	0.05 ± 0.07	nd
C9Z2N2	SCAB_57161	Endo-β-1.6-galactanase	0.07 ± 0.03	0.36 ± 0.11	0.35 ± 0.04	nd	nd	0.02 ± 0.02
C9Z451	SCAB_57751	Cellobiose-binding transport system	nd	0.11 ± 0.07	0.18 ± 0.06	nd	0.02 ± 0.02	nd
C9ZDW4	SCAB_63891	ABC-type xylose transport system	0.13 ± 0.10	0.63 ± 0.14	0.34 ± 0.18	0.03 ± 0.04	1.00 ± 0.30	0.75 ± 0.15
C9ZFW2	SCAB_66021	β-xylosidase	0.09 ± 0.02	0.23 ± 0.19	0.23 ± 0.18	nd	0.01 ± 0.02	0.01 ± 0.02
C9ZFW3	SCAB_66031	Arabinofuranosidase	0.46 ± 0.06	0.73 ± 0.26	0.67 ± 0.14	0.03 ± 0.03	0.32 ± 0.15	0.26 ± 0.12
C9YY63	SCAB_69711^d^	Phosphoglycerate kinase	0.07 ± 0.05	0.02 ± 0.02	0.00 ± 0.01	0.02 ± 0.02	nd	nd
C9Z2V1	SCAB_72711	Endo β-1.4-xylanase	0.29 ± 0.12	0.20 ± 0.11	0.25 ± 0.05	nd	nd	0.05 ± 0.05
C9Z4J7	SCAB_74141	α-N-furanosidase	0.34 ± 0.14	0.19 ± 0.11	0.20 ± 0.11	nd	0.01 ± 0.01	0.03 ± 0.02
C9ZB22	SCAB_77441	α-arabinanase	0.06 ± 0.01	0.05 ± 0.01	0.06 ± 0.02	0.01 ± 0.02	0.08 ± 0.02	0.11 ± 0.02
C9ZCR4	SCAB_78891	Glycosyl hydrolase	0.08 ± 0.06	0.28 ± 0.16	0.16 ± 0.06	nd	0.00 ± 0.01	0.11 ± 0.06
C9ZE94	SCAB_79241	Arabinofuranosidase	0.60 ± 0.16	1.90 ± 0.61	1.51 ± 0.68	nd	0.25 ± 0.11	0.07 ± 0.06
C9ZE95	SCAB_79251	Xylanase A	0.79 ± 0.30	3.26 ± 1.60	3.17 ± 1.14	0.04 ± 0.01	0.13 ± 0.06	0.12 ± 0.06
C9Z041	SCAB_84801	Arabinase	0.17 ± 0.06	0.46 ± 0.20	0.45 ± 0.15	0.03 ± 0.06	0.61 ± 0.11	0.47 ± 0.11
C9Z1I5	SCAB_85231	Chitinase	0.17 ± 0.02	0.80 ± 0.13	0.46 ± 0.16	nd	0.42 ± 0.19	0.26 ± 0.16
C9Z351	SCAB_86311	Endoglucanase	nd	0.29 ± 0.16	0.27 ± 0.21	nd	0.01 ± 0.02	0.02 ± 0.03
C9Z674	SCAB_88071	Arabinofuranosidase	0.05 ± 0.04	0.14 ± 0.04	0.14 ± 0.06	nd	0.05 ± 0.05	0.02 ± 0.03
C9Z804	SCAB_89741	Cellulose-binding protein	0.33 ± 0.09	0.96 ± 0.23	0.55 ± 0.12	nd	0.33 ± 0.14	0.24 ± 0.03
C9Z9L5	SCAB_90081	Cellulase B	0.21 0.08	0.21 0.08	0.11 ± 0.03	nd	0.03 0.04	0.03 0.03
C9Z9L6	SCAB_90091	Cellulase	0.30 ± 0.09	0.85 ± 0.30	0.67 ± 0.22	nd	0.07 ± 0.06	0.06 ± 0.04
C9Z9L7	SCAB_90101	Cellulase	0.59 ± 0.21	1.03 ± 0.39	0.75 ± 0.40	nd	0.56 ± 0.47	0.19 ± 0.07
Amino acid metabolism								
C9Z1W0	SCAB_9531^d^	Catalase-peroxidase	0.10 ± 0.01	0.06 ± 0.06	0.04 ± 0.05	0.06 ± 0.05	nd	nd
C9Z862	SCAB_13321	X-prolyl-dipeptidyl aminopeptidase	0.21 ± 0.16	0.28 ± 0.02	0.18 ± 0.09	0.02 ± 0.03	nd	nd
C9ZGG7	SCAB_18081	γ-glutamyltranspeptidase	0.39 ± 0.06	0.32 ± 0.08	0.32 ± 0.08	0.01 ± 0.01	nd	0.02 ± 0.02
C9ZGM2	SCAB_18661	Serine protease	0.19 ± 0.17	nd	nd	0.01 ± 0.02	0.17 ± 0.16	nd
C9Z6V3	SCAB_27921	Neutral zinc metalloprotease	0.23 ± 0.12	0.04 ± 0.02	0.04 ± 0.01	0.01 ± 0.02	0.03 ± 0.03	nd
C9ZAN4	SCAB_46111	Single-stranded DNA-binding protein	0.16 ± 0.05	0.26 ± 0.12	0.29 ± 0.09	0.04 ± 0.06	0.05 ± 0.05	0.11 ± 0.05
C9YTK4	SCAB_51101^d^	Phosphoserine aminotransferase	0.14 ± 0.09	0.41 ± 0.23	0.50 ± 0.16	0.06 ± 0.05	0.09 ± 0.05	0.07 ± 0.06
C9Z498	SCAB_58251	Tripeptidyl aminopeptidase	0.26 ± 0.03	0.10 ± 0.06	0.08 ± 0.05	0.10 ± 0.11	0.06 ± 0.05	0.01 ± 0.01
C9ZHG5	SCAB_66881^d^	Glutamine synthetase	0.05 ± 0.04	0.30 ± 0.09	0.09 ± 0.05	0.01 ± 0.02	nd	0.03 ± 0.01
C9YY61	SCAB_69691	Zinc-binding carboxypeptidase	0.16 ± 0.04	0.05 ± 0.01	0.04 ± 0.02	0.02 ± 0.01	0.07 ± 0.02	0.05 ± 0.02
C9YZP9	SCAB_70761	Solute-binding protein	0.11 ± 0.05	0.46 ± 0.22	0.35 ± 0.17	nd	0.43 ± 0.19	0.29 ± 0.08
C9Z1E7	SCAB_72231	Serine protease	0.28 ± 0.15	0.58 ± 0.20	0.50 ± 0.13	0.01 ± 0.02	0.25 ± 0.07	0.24 ± 0.12
C9Z1N0	SCAB_85681^d^	Tyrosinase	0.31 ± 0.05	0.02 ± 0.03	0.01 ± 0.01	0.01 ± 0.02	0.01 ± 0.02	nd
Nucleotide metabolism								
C9ZGX4	SCAB_49491	5'-nucleotidase	0.17 ± 0.07	0.12 ± 0.05	0.06 ± 0.04	0.01 ± 0.01	nd	nd
Inorganic ion metabolism								
C9ZH91	SCAB_66141	Alkaline phosphatase	0.21 ± 0.07	0.25 ± 0.03	0.18 ± 0.08	nd	nd	0.03 ± 0.03
General function prediction only								
C9Z871	SCAB_13411	Oxidoreductase	1.04 ± 0.30	0.87 ± 0.20	0.70 ± 0.28	0.22 ± 0.16	0.16 ± 0.21	0.02 ± 0.03
C9ZAW6	SCAB_62471	Aminopeptidase	0.30 ± 0.07	0.54 ± 0.28	0.43 ± 0.17	0.01 ± 0.01	0.19 ± 0.14	0.02 ± 0.02
C9ZE96	SCAB_79261	Feruloyl esterase	0.30 ± 0.16	0.48 ± 0.29	0.41 ± 0.20	nd	0.03 ± 0.04	0.03 ± 0.02
Unknown function								
C9YVL7	SCAB_5681		0.44 ± 0.20	0.61 ± 0.19	0.54 ± 0.31	nd	0.11 ± 0.14	nd
C9YUN3	SCAB_20171		0.11 ± 0.11	0.08 ± 0.06	0.09 ± 0.03	nd	0.09 ± 0.05	0.03 ± 0.05
C9ZGQ1	SCAB_33981		1.19 ± 0.44	3.88 ± 1.18	2.99 ± 0.95	0.18 ± 0.09	1.95 ± 0.48	1.33 ± 0.32
C9YUT0	SCAB_35731		0.14 ± 0.05	nd	nd	nd	nd	0.06 ± 0.05
C9Z411	SCAB_41931		0.07 ± 0.12	nd	nd	nd	0.07 ± 0.12	nd
C9ZHG0	SCAB_66831		0.09 ± 0.04	0.12 ± 0.07	0.09 ± 0.11	0.09 ± 0.00	0.01 ± 0.03	nd
C9Z4J0	SCAB_74081		0.30 ± 0.06	0.44 ± 0.32	0.32 ± 0.17	nd	0.02 ± 0.04	0.01 ± 0.01
C9Z7P8	SCAB_75721		0.29 ± 0.04	0.34 ± 0.08	0.24 ± 0.09	nd	0.01 ± 0.02	0.10 ± 0.07

^a^Uniprot accession number; ^b^Data are the mean of three replicates; ^c^nd: not detected; ^d^Protein with intracellular localisation prediction.

**Table 2:** **Proteins specifically produced by *Streptomyces scabiei *** **in the casein-suberin medium**

**Protein assignation**^**a**^	**Gene assignation**	**Putative function**	**Abundance (spectrum count/ MW**^**b**^**)**		
			**Day 1**	**Day 3**	**Day 5**
Translational, ribosomal structure and biogenesis					
C9Z241	SCAB_25261^d^	30S ribosomal protein S15	0.15 ± 0.26	0.38 ± 0.27	0.03 ± 0.05
C9YW50	SCAB_36661^d^	50S ribosomal protein L17	0.43 ± 0.22	0.51 ± 0.32	0.40 ± 0.32
C9YW52	SCAB_36681^d^	30S ribosomal protein S11	0.31 ± 0.18	0.08 ± 0.11	nd
C9YW59	SCAB_36751^d^	50S ribosomal protein L15	0.21 ± 0.18	0.42 ± 0.27	0.21 ± 0.20
C9YW62	SCAB_36781^d^	50S ribosomal protein L18	0.31 ± 0.18	0.32 ± 0.11	0.24 ± 0.14
C9YW66	SCAB_36821^d^	50S ribosomal protein L5	0.38 ± 0.21	0.33 ± 0.11	0.13 ± 0.05
C9YW67	SCAB_36831^d^	50S ribosomal protein L24	0.06 ± 0.10	0.03 ± 0.04	nd
C9YW69	SCAB_36851^d^	30S ribosomal protein S17	0.15 ± 0.10	0.38 ± 0.33	0.05 ± 0.08
C9YW71	SCAB_36871^d^	50S ribosomal protein L16	0.15 ± 0.13	0.08 ± 0.10	0.01 ± 0.03
C9YW75	SCAB_36911	50S ribosomal protein L2	0.40 ± 0.28	0.97 ± 0.24	0.60 ± 0.12
C9YW76	SCAB_36921	50S ribosomal protein L23	0.24 ± 0.10	0.32 ± 0.19	0.29 ± 0.22
C9YW79	SCAB_36951^d^	30S ribosomal protein S10	0.36 ± 0.05	0.32 ± 0.08	nd
C9YW95	SCAB_37121	30S ribosomal protein S12	0.24 ± 0.11	0.14 ± 0.09	0.05 ± 0.07
C9Z0W3	SCAB_39971^d^	30S ribosomal protein S18 1	nd	0.07 ± 0.18	nd
C9Z0W7	SCAB_40011^d^	50S ribosomal protein L28 2	nd	0.29 ± 0.10	0.17 ± 0.10
C9ZAN3	SCAB_46101^d^	30S ribosomal protein S6	0.09 ± 0.16	0.23 ± 0.13	0.11 ± 0.09
C9Z2I8	SCAB_56731^d^	Ribonuclease PH	0.04 ± 0.04	0.10 ± 0.03	0.11 ± 0.03
C9Z7H2	SCAB_60151^d^	50S ribosomal protein L21	0.33 ± 0.08	0.36 ± 0.31	0.25 ± 0.33
C9Z7H3	SCAB_60161^d^	50S ribosomal protein L27	0.22 ± 0.11	0.48 ± 0.15	0.19 ± 0.15
C9YWQ2	SCAB_69061^d^	30S ribosomal protein S1	0.07 ± 0.06	nd	nd
C9Z316	SCAB_73401^d^	tRNA (adenine-N(1)-)-methyltransferase	0.02 ± 0.03	0.07 ± 0.07	0.07 ± 0.04
C9Z4H9	SCAB_73971	50S ribosomal protein L35	0.57 ± 0.00	0.90 ± 0.44	0.48 ± 0.28
C9Z4I0	SCAB_73981^d^	50S ribosomal protein L20	0.38 ± 0.35	0.42 ± 0.25	0.30 ± 0.13
Transcription					
C9Z8G5	SCAB_28931^d^	Transcription termination factor	0.03 ± 0.04	nd	nd
C9YWA1	SCAB_37181^d^	DNA-directed RNA polymerase subunit β	nd	0.01 ± 0.02	nd
C9YWA2	SCAB_37191^d^	DNA-directed RNA polymerase subunit β	nd	0.02 ± 0.02	0.01 ± 0.01
Posttranslational modification, protein turnover, chaperones					
C9YYX4	SCAB_23161^d^	Membrane protease	nd	0.04 ± 0.06	0.10 ± 0.07
C9YYJ3	SCAB_84021^d^	Carbamoyltransferase	nd	0.14 ± 0.11	0.17 ± 0.07
Cell wall/membrane/envelope biogenesis					
C9Z1V1	SCAB_9441^d^	Sugar isomerase	nd	0.00 ± 0.01	0.02 ± 0.02
C9Z234	SCAB_25191^d^	Dihydrodipicolinate synthase	0.02 ± 0.04	0.01 ± 0.03	0.04 ± 0.05
C9ZAN0	SCAB_46061^d^	Alanine racemase	0.01 ± 0.02	0.01 ± 0.02	0.01 ± 0.02
C9Z2W0	SCAB_72801	Glycosyl hydrolase	0.28 ± 0.15	0.90 ± 0.20	0.83 ± 0.12
Signal transduction mechanism					
C9YYC2	SCAB_70311^d^	TerD-like stress protein	nd	0.01 ± 0.02	0.04 ± 0.03
Defense mechanism					
C9ZAA4	SCAB_29931^d^	Nickel superoxide dismutase	0.16 ± 0.08	nd	0.01 ± 0.03
C9YZ91	SCAB_38731^d^	β-lactamase	0.63 ± 0.05	0.54 ± 0.07	0.34 ± 0.11
C9Z5I1	SCAB_42661^d^	β-lactamase	0.10 ± 0.02	nd	nd
C9ZGZ0	SCAB_49661^d^	Peroxiredoxin	nd	0.03 ± 0.06	0.09 ± 0.11
C9Z043	SCAB_84821	β-lactamase	0.02 ± 0.03	0.18 ± 0.04	0.11 ± 0.05
Secondary metabolites biosynthesis, transport and catabolism					
C9YYT5	SCAB_8041	Aminohydrolase	0.01 ± 0.02	0.26 ± 0.11	0.22 ± 0.11
C9YWT5	SCAB_69391^d^	Pseudouridine-5'-phosphate glycosidase	nd	0.03 ± 0.05	0.02 ± 0.03
C9YU69	SCAB_82441	Poly(3-hydroxyalkanoate) depolymerase C	nd	0.01 ± 0.01	0.03 ± 0.03
C9Z6A5	SCAB_88391^d^	2-hydroxyhepta-2.4-diene-1.7-dioate isomerase	nd	nd	0.04 ± 0.05
Energy production and conversion					
C9ZBX1	SCAB_31271^d^	Ferredoxin	0.10 ± 0.04	nd	nd
C9YU38	SCAB_82111^d^	Oxidoreductase	0.03 ± 0.04	0.02 ± 0.03	0.01 ± 0.02
Lipid metabolism					
C9ZD66	SCAB_16601^d^	CoA transferase	nd	nd	0.01 ± 0.02
C9Z6Y2	SCAB_28231^d^	Acetate/propionate kinase	nd	0.01 ± 0.02	0.04 ± 0.04
C9ZGV4	SCAB_34521^d^	Enoyl-CoA hydratase	0.03 ± 0.03	nd	nd
C9Z776	SCAB_44071^d^	Esterase-lipase	0.18 ± 0.05	0.10 ± 0.07	0.07 ± 0.02
C9YTK3	SCAB_51091	Esterase-lipase	nd	0.14 ± 0.05	0.13 ± 0.05
C9YY49	SCAB_54571^d^	Acetyl CoA acyl transferase	0.01 ± 0.01	0.10 ± 0.09	0.14 ± 0.07
C9YYE5	SCAB_70541	Lipolytic enzyme	0.07 ± 0.09	nd	nd
C9Z7Q3	SCAB_75771^d^	Acyl-CoA dehydrogenase	0.02 ± 0.01	0.02 ± 0.03	nd
Degradation of aromatic compounds					
C9Z2P6	SCAB_57301	3-oxo-5.6-dehydrosuberyl-CoA semialdehyde dehydrogenase	0.13 ± 0.17	nd	nd
Carbohydrate metabolism					
C9YSY4	SCAB_4561	Glycosyl hydrolase	0.00 ± 0.00	0.02 ± 0.02	0.00 ± 0.00
C9YVM5	SCAB_5761	Glycosyl hydrolase	nd	0.04 ± 0.05	0.01 ± 0.01
C9YVN4	SCAB_5861	Carbohydrate esterase	nd	0.01 ± 0.01	0.06 ± 0.06
C9YVP5	SCAB_5981	Cellulase B	0.09 ± 0.06	0.31 ± 0.19	0.20 ± 0.13
C9YVP8	SCAB_6011	Glycosyl hydrolase/xylanase	0.01 ± 0.01	nd	0.03 ± 0.04
C9YX59	SCAB_6471	α-L-fucosidase	nd	0.09 ± 0.11	0.06 ± 0.09
C9Z1U5	SCAB_9381	Exo-α-sialidase	0.16 ± 0.08	0.34 ± 0.17	0.37 ± 0.14
C9Z885	SCAB_13561	Glycosyl hydrolase	0.02 ± 0.03	0.01 ± 0.01	0.00 ± 0.01
C9ZBJ5	SCAB_15481^d^	α-mannosidase	nd	0.04 ± 0.03	0.07 ± 0.07
C9ZD61	SCAB_16551	Mannosidase	0.02 ± 0.04	0.18 ± 0.14	0.15 ± 0.12
C9ZD71	SCAB_16651	SugarP isomerase	0.01 ± 0.02	0.05 ± 0.03	0.02 ± 0.02
C9ZEQ1	SCAB_17021	Cellulase	0.04 ± 0.02	0.47 ± 0.13	0.36 ± 0.10
C9ZEQ3	SCAB_17041	Glycosyl transferase	0.02 ± 0.03	nd	nd
C9Z574	SCAB_26801	Polysaccharide lyase	nd	0.04 ± 0.03	0.05 ± 0.04
C9YUZ7	SCAB_36421^d^	β-xylosidase	nd	0.05 ± 0.02	0.05 ± 0.02
C9YW88	SCAB_37051	Cellulase/xylanase	0.09 ± 0.03	0.24 ± 0.09	0.35 ± 0.14
C9Z725	SCAB_43531	Polysaccharide lyase	0.00 ±	0.02 ± 0.03	0.04 ± 0.05
C9Z789	SCAB_44201	β-galactosidase	0.01 ± 0.02	0.02 ± 0.02	0.00 ± 0.00
C9Z4A0	SCAB_58271	Glycosyl hydrolase	nd	0.01 ± 0.01	0.02 ± 0.03
C9ZDV4	SCAB_63781^d^	α-L-arabinofuranosidase	nd	0.01 ± 0.01	0.04 ± 0.04
C9YYE3	SCAB_70521	Bifunctional pectate lyase/pectinesterase	0.02 ± 0.02	nd	nd
C9YYE6	SCAB_70551	Pectate lyase	0.91 ± 0.12	0.01 ± 0.01	nd
C9YYE7	SCAB_70561	Pectinesterase	0.30 ± 0.04	0.05 ± 0.02	0.03 ± 0.03
C9YYE8	SCAB_70571	Pectinesterase	0.24 ± 0.08	0.07 ± 0.02	0.04 ± 0.03
C9YYF0	SCAB_70591	Pectate lyase	0.36 ± 0.09	0.41 ± 0.18	0.29 ± 0.30
C9Z2Z4	SCAB_73161	Secreted protein	0.36 ± 0.06	0.17 ± 0.17	0.04 ± 0.06
C9Z623	SCAB_74681	Licheninase	0.07 ± 0.07	0.34 ± 0.07	0.27 ± 0.11
C9ZAZ8	SCAB_77201	Glycosyl hydrolase	0.12 ± 0.08	0.95 ± 0.25	0.71 ± 0.14
C9ZB17	SCAB_77391	Glycosyl hydrolase	0.01 ± 0.02	0.10 ± 0.11	0.06 ± 0.06
C9ZE74	SCAB_79011	Acetyl-xylan esterase	nd	0.59 ± 0.13	0.52 ± 0.09
C9ZEB7	SCAB_79481	Xylanase A	0.01 ± 0.02	0.02 ± 0.03	0.02 ± 0.03
C9ZFY5	SCAB_79861^d^	Xylose isomerase	0.04 ± 0.01	0.10 ± 0.06	0.11 ± 0.05
C9YU11	SCAB_81841	Glycosyl hydrolase	0.13 ± 0.11	0.06 ± 0.06	0.01 ± 0.01
C9YU29	SCAB_82021	β-mannosidase	nd	0.22 ± 0.06	0.24 ± 0.07
C9YU66	SCAB_82411	Pectate lyase	nd	0.07 ± 0.08	0.06 ± 0.07
C9Z820	SCAB_89901^d^	β-D-xylosidase	nd	0.02 ± 0.02	0.02 ± 0.02
Amino acid metabolism					
C9YVT3	SCAB_6381	Extracellular small neutral protease	0.25 ± 0.21	0.08 ± 0.11	0.05 ± 0.08
C9YYP7	SCAB_7651	Zinc metalloprotease	0.13 ± 0.05	0.04 ± 0.04	0.03 ± 0.02
C9YYX3	SCAB_23151^d^	Arginine deaminase	nd	0.00 ± 0.01	0.03 ± 0.02
C9Z238	SCAB_25231^d^	Dihydrodipicolinate reductase	nd	0.03 ± 0.05	0.01 ± 0.03
C9Z281	SCAB_25691^d^	4-aminobutyrate aminotransferase	0.01 ± 0.03	0.04 ± 0.04	0.02 ± 0.01
C9Z5A0	SCAB_27061^d^	Ketol-acid reductoisomerase	0.02 ± 0.02	0.02 ± 0.02	0.00 ± 0.01
C9YTF8	SCAB_35641^d^	Serine hydroxymethyltransferase 4	0.02 ± 0.02	nd	0.01 ± 0.01
C9Z8U5	SCAB_45071^d^	Zinc aminopeptidase	0.04 ± 0.01	0.05 ± 0.01	0.07 ± 0.03
C9ZC37	SCAB_46731^d^	Xaa-Pro aminopeptidase	0.01 ± 0.01	0.06 ± 0.04	0.16 ± 0.12
C9Z7B6	SCAB_59611^d^	Aminopeptidase N	0.05 ± 0.04	nd	nd
C9Z4N7	SCAB_87271	Peptidase	0.03 ± 0.05	0.10 ± 0.13	0.04 ± 0.04
Coenzyme metabolism					
C9Z1Y4	SCAB_9771^d^	Cobalamin biosynthesis protein	0.02 ± 0.02	0.18 ± 0.04	0.12 ± 0.07
C9Z3M4	SCAB_25861^d^	Aminotransferase	nd	0.00 ± 0.01	0.03 ± 0.03
C9Z402	SCAB_41841^d^	Aminotransferase	nd	0.00 ± 0.01	0.01 ± 0.03
C9Z638	SCAB_74841^d^	Pyridoxal biosynthesis lyase pdxS	0.03 ± 0.05	nd	nd
C9Z7L0	SCAB_75311^d^	S-adenosylmethionine synthase	nd	0.01 ± 0.02	0.02 ± 0.02
Nucleotide metabolism					
C9Z407	SCAB_41891^d^	Adenylosuccinate synthetase	0.01 ± 0.03	nd	nd
C9ZDR6	SCAB_47861^d^	Phosphoribosylformylglycinamidine cyclo-ligase	0.01 ± 0.02	0.00 ± 0.01	0.03 ± 0.02
C9Z7F8	SCAB_60011^d^	Nucleoside diphosphate kinase	0.02 ± 0.04	0.08 ± 0.09	0.09 ± 0.13
C9YVK8	SCAB_68841	5' nucleotidase	0.03 ± 0.02	0.15 ± 0.09	0.08 ± 0.07
Inorganic ion metabolism					
C9YVE4	SCAB_68191^d^	Alkaline phosphatase	0.13 ± 0.04	0.06 ± 0.04	0.06 ± 0.05
C9ZB70	SCAB_77971	Alkaline phosphatase	0.16 ± 0.01	0.19 ± 0.07	0.17 ± 0.11
General function prediction only					
C9YSY7	SCAB_4591	Acyltransferases	0.07 ± 0.07	0.02 ± 0.03	nd
C9YVP7	SCAB_6001^d^	Feruloyl esterase	nd	0.05 ± 0.04	0.06 ± 0.06
C9Z3D2	SCAB_10131	Glycosyl hydrolase	nd	0.06 ± 0.03	0.06 ± 0.03
C9YT61	SCAB_19541	Carbohydrate esterase	0.02 ± 0.03	0.05 ± 0.03	0.01 ± 0.02
C9Z233	SCAB_25181^d^	B lactamase	0.01 ± 0.01	0.03 ± 0.04	0.05 ± 0.03
C9Z6S2	SCAB_27611^d^	Phage tail sheath protein FI	nd	0.03 ± 0.01	0.09 ± 0.05
C9Z8S0	SCAB_44821^d^	Phosphatase	0.03 ± 0.05	0.00 ± 0.01	nd
C9Z994	SCAB_61611^d^	Protease	0.05 ± 0.04	0.14 ± 0.03	0.03 ± 0.03
C9ZAQ7	SCAB_61831^d^	Metalloendopeptidase	nd	nd	0.03 ± 0.03
C9ZAU3	SCAB_62211	Phosphatase	nd	0.11 ± 0.05	0.17 ± 0.07
C9ZDW7	SCAB_63921^d^	Dehydratase	nd	0.04 ± 0.05	0.07 ± 0.03
C9ZHB0	SCAB_66321^d^	F420-dependent NADP reductase	nd	0.02 ± 0.03	0.05 ± 0.05
C9ZB18	SCAB_77401	Glycosyl hydrolase	nd	0.06 ± 0.03	0.05 ± 0.04
C9ZCL9	SCAB_78431	Tripeptidylaminopeptidase	0.13 ± 0.01	0.14 ± 0.04	0.14 ± 0.11
C9ZEA8	SCAB_79391	Glycosyl hydrolase	nd	0.13 ± 0.07	0.11 ± 0.09
C9ZEC6	SCAB_79571	Glycosyl hydrolase	nd	0.03 ± 0.03	0.01 ± 0.02
C9YYG1	SCAB_83671^d^	Acetyl esterase	nd	0.00 ± 0.01	0.02 ± 0.05
C9Z047	SCAB_84861	Amidase	0.21 ± 0.05	0.11 ± 0.03	0.06 ± 0.06
Unknown function					
C9Z6Q2	SCAB_12841		0.44 ± 0.12	0.70 ± 0.25	0.59 ± 0.17
C9ZBK5	SCAB_15581^d^		0.04 ± 0.04	0.03 ± 0.02	0.03 ± 0.03
C9YVU0	SCAB_20641		0.07 ± 0.04	0.34 ± 0.09	0.38 ± 0.11
C9Z0X8	SCAB_40131		0.28 ± 0.13	0.14 ± 0.05	0.07 ± 0.03
C9Z5E6	SCAB_42301^d^		nd	0.03 ± 0.06	0.03 ± 0.06
C9ZGX5	SCAB_49501		0.06 ± 0.06	nd	nd
C9YV55	SCAB_52141^d^		0.02 ± 0.03	0.03 ± 0.03	0.08 ± 0.09
C9Z1G8	SCAB_72441		0.17 ± 0.06	0.27 ± 0.09	0.32 ± 0.10
C9Z609	SCAB_74541		0.12 ± 0.14	nd	0.02 ± 0.04
C9ZG06	SCAB_80071		0.02 ± 0.03	nd	nd

^a^Uniprot accession number; ^b^Data are the mean of three replicates; ^c^nd: not detected; ^d^Protein with intracellular localisation prediction.

**Table 3:** **Distribution of *Streptomyces scabiei *** **proteins into functional groups**

**Class of proteins**	**Number of proteins**		
	**In CM only**	**Overproduced in CSM**^**a**^	**In CSM only**
Translational, ribosomal structure and biogenesis	2^b^	17^b^	23^b^
Transcription	2	1^b^	3^b^
Posttranslational modification, protein turnover, chaperone	11	0	2^b^
Cell wall/membrane/envelope biogenesis	14	2	4^b^
Signal transduction mechanisms	3^b^	0	1^b^
Secretion	3	0	0
Defense mechanism and virulence	3	2	5^b^
Secondary metabolites biosynthesis, transport and catabolism	0	0	4
Differentiation	1	1	0
Energy production and conversion	4	4^b^	2^b^
Lipid metabolism	1	3	8^b^
Degradation of aromatic compounds	0	0	1
Carbohydrate metabolism	5	45	36
Amino acid metabolism	14	13	11^b^
Coenzyme metabolism	1^b^	0	5
Nucleotide metabolism	1^b^	1	4^b^
Inorganic ion metabolism	3	1	2
General function prediction only	9	3	18
Unknown function	47	8	10

^a^Protein with a spectral counting/MW 5-fold greater in CSM than in CM; ^b^Most or all proteins have a predicted intracellular localisation.

Bradford protein assays estimated supernatant protein concentrations in CM to be 280 ± 113, 275 ± 33 and 217 ± 10 μg/mL in 1-, 3- and 5-day-old cultures, respectively. In CSM, protein concentrations were estimated to be 361 ± 45, 350 ± 13 and 285 ± 9 μg/mL in 1-, 3- and 5-day-old cultures, respectively. This higher production of extracellular proteins in CSM may reflect the recalcitrant nature of suberin [6]. Because of the complexity of plant cell walls, some microorganisms secrete up to 50% of their total protein during growth on such a substrate [14]. Although the majority of the proteins (69.3%) found in the supernatants were predicted to have an extracellular localisation by SignalP, SecretomeP, TatFind or TatP analysis, some proteins with a predicted intracellular location were also identified. The proportion of predicted intracellular proteins recovered in the 3- and 5-day-old culture supernatants was higher in CSM than in CM samples (Table 4). A higher concentration of predicted intracellular proteins in CSM may reflect contamination of the secretome by intracellular content of lysed cells. Most of the proteins included in translational, ribosomal structure and biogenesis, coenzyme metabolism and nucleotide metabolism classes were predicted to be localized in the cytoplasm and were found predominantly in samples grown in CSM (Table 3). Higher levels of lysis in CSM may be due to the fact that monomers associated with the suberin structure act as membrane perturbants [15]. Furthermore, suberin has been shown to increase membrane fluidity in *S. scabiei*[6].

**Table 4:** **Proportion of predicted extracellular proteins in the secretome *Streptomyces scabiei *** **grown in CM of CSM**

**Cultivation time**	**Proportion of predicted extracellular proteins (%)**	
	**CM**	**CSM**
1-day-old culture	73	68
3-day-old culture	87	65
5-day-old culture	88	66

Although both CM and CSM contain casein as carbon and nitrogen sources, 124 different proteins were specifically detected in the CM supernatants, indicating that the presence of suberin might repress expression of several genes (Additional file 2: Table S2). As reported in other proteomic studies [2,3], a non-negligible number of detected proteins have unknown functions and surprisingly, several proteins of unknown function were associated with growth in CM (Table 3). Proteins included in posttranscriptional modification, protein turnover and chaperones and in cell envelope biogenesis classes were also found in higher proportion in CM-grown samples (Table 3). It has been reported that suberin induces a thickening of the cell wall [6] and the differential production of proteins involved in cell envelope biogenesis suggest that both cell wall structure and composition of *S. scabiei* may differ depending on the culture media.

Among the 246 proteins associated with both culture media, 41% of them were 5-fold more abundant in CSM after 1, 3 or 5 days of growth (Table 1). A total of 139 proteins were specifically detected in CSM supernatants indicating that the presence of suberin triggers the expression of a number of genes (Table 2). Several of the proteins found in higher proportion in the presence of suberin were classified into the carbohydrate metabolism class and two functional groups of proteins (degradation of aromatic compounds and secondary metabolites biosynthesis, transport and catabolism) were exclusively associated with growth in CSM (Table 3).

In addition, a factor C-like morphological differentiation protein (C9Z7Z7) was more abundant and was detected at an earlier time point in the *S. scabiei* secretome when the bacterium was grown in the presence of suberin. Promotion of differentiation by suberin in the genus *Streptomyces* has been previously reported [6]. This promotion might be at least partly due to a higher concentration of the secreted factor C, which is known to play a role in morphological differentiation and to restore wild-type developmental gene expression to an A-factor non-producing mutant of *Streptomyces griseus*[16]. Furthermore, a factor C null mutant of strain *Streptomyces albidoflavus*, a common scab-inducing strain, exhibited a bald phenotype and appeared less pathogenic than the wild-type bacteria [17]. Suberin seemed also to promote the initiation of the secondary metabolism that triggered the production of thaxtomin A, a phytotoxin essential for *S. scabiei* pathogenicity [5].

### Abundance of extracellular proteins associated with carbohydrate metabolism in suberin-containing medium

Out of 240 proteins overproduced or exclusively produced in the presence of suberin, 81 (33%) were involved in carbohydrate metabolism (Tables 1 and 2) and 49 of these were identified as glycosyl hydrolases (GH) using CAZy classification (Table 3). Two proteins (C9ZE95 and C9Z5L1) in this class figured among the ten most abundant proteins in the 24-h CSM supernatant. After 5 days of incubation, these two proteins as well as three other proteins of the same class (C9ZD50, C9ZEP9 and C9ZE94) were included in the ten most highly represented proteins in the CSM supernatant. In contrast, no proteins belonging to the carbohydrate metabolism class appeared among the ten most abundant proteins in the CM at any sampling time. This abundance of glycosyl hydrolases in CSM was unexpected considering that this culture medium was not supplemented with polysaccharides. Nevertheless, some of the putative glycosyl hydrolases present in the supernatant were active, since CSM supernatants exhibited cellulase, xylanase and licheninase activities (Table 5).

**Table 5:** **Enzymatic activities (mU/ml)**^**a **^**associated with supernatants of *Streptomyces scabiei *** **grown in CM or CSM**

	**Xylanase**		**Cellulase**		**Licheninase**	
	**CM**	**CSM**	**CM**	**CSM**	**CM**	**CSM**
1-day-old culture	0	30.1 ± 7.4	0	12.2 ± 2.1	5.7 ± 2.7	30.8 ± 23.6
3-day-old culture	0	314.0 ± 11.6	0	26.3 ± 4.3	5.1 ± 3.8	50.2 ± 2.5
5-day-old culture	9.7 ± 2.5	372.0 ± 9.0	7.7 ± 0.3	32.8 ± 2.1	64.1 ± 4.9	116.0 ± 4.5

^a^Data are the mean of three replicates.

Production of glycosyl hydrolases in the presence of suberin may be due to the presence of sugar contaminants in suberin. The polymer is anchored in the plant cell wall and is tightly associated with other cell wall components such as polysaccharides [10]. Enzymatic and extractive protocols have been optimized to remove around 95% of the unsuberized cell walls and waxes from suberized potato periderm [18]. Nevertheless, cell wall polysaccharides are covalently attached to the polyester biopolymer and could thus be inaccessible to enzymes used to purify suberin. When grown in the presence of suberin, contaminating cell wall polysaccharides represent a higher carbon energy supply for the bacteria than the aliphatic and aromatic fractions of suberin, explaining the importance of this group of enzymes in *S. scabiei* secretome.

A set of enzymes possibly involved in xylan catabolism were specific to the suberin-containing medium or were found in higher proportion in the presence of suberin. A putative xylanase A (C9ZE95) was among the most abundant proteins detected in suberin-containing medium at all sampling times and its abundance was between 20 to 26 times higher than in CM samples (Table 1). Complete hydrolysis of xylan requires xylanases such as endo-β-1, 4-xylanases, β-xylosidase and other enzymes that cleave side chain sugars from the xylan backbone, such as α-arabinofuranosidases and acetyl esterases, for example. Most xylanases found in this study belong to glycoside hydrolase families GH5, GH8, GH10, GH11, GH30 and GH43 (CAZy classification [19]).

Some of the proteins detected only in CSM and included in the carbohydrate metabolism class were putative polysaccharide lyases (C9YU66, C9YYE6, C9YYF0, C9Z574 and C9Z725) or carbohydrate esterases (C9YVN4, C9YVP5, C9YYE7, C9YYE8 and C9ZE74). Among them, proteins C9YYE6, C9YYE7 and C9YYE8, encoded by three adjacent genes, were predicted to belong to an operon of four genes (http://www.microbesonline.org/operons/gnc680198.html) and are probably involved in pectin degradation. The fourth gene of the operon encodes for the lipolytic enzyme C9YYE5, which was also only detected in suberin-containing medium.

In addition to enzymes involved in xylan and pectin degradation, other types of polysaccharide-degrading enzymes were detected specifically in CSM: cellulases (C9YVP5, C9YW88 and C9ZEQ1), a putative licheninase (C9Z623) and several enzymes involved in the hydrolysis of hemicellulose compounds. Glycosyl hydrolase activity in CS and CSM supernatants has been assayed on cellulose, xylan and lichenin and our experiments revealed that CSM supernatants possessed higher cellulase, xylanase and licheninase activity (Table 5). Furthermore, addition of a small amount of suberin to *S. scabiei* culture media containing carboxymethyl cellulose or xylan as the main carbon source considerably increased the cellulase and xylanase activities, respectively (unpublished data). Given that the amount of suberin added in the culture media is relatively small, the increase in enzymatic activity is unlikely to be attributable to contamination of the suberin polymer with cellulose or xylan. This increase might be due to the secretion of glycosyl hydrolases specifically induced by the presence of suberin or to an overproduction of extracellular enzymes caused by the addition of suberin. Phenolic suberin compounds might be partly responsible for the high glycosyl hydrolase activity since various phenolic compounds such as gallic acid, tannic acid, maleic acid and salicylic acid were shown to induce expression of various genes encoding cellulases [20]. The promotion of secondary metabolism by suberin [6] could also explain this overproduction as the A-factor regulon includes many extracellular glycosyl hydrolases in *S. griseus*[21].

Topochemical studies have shown that a part of the suberin polyaromatic domain is located in the primary and tertiary cell walls [10]. Polyaromatic compounds from suberin are thus associated with polysaccharide-type glycosides but the nature of their covalent link remains speculative [10]. The fact that several secreted carbohydrate esterases identified in this study belonged to carbohydrate esterase families CE1, CE2, CE7 and CE12 (Table 3) that include acetyl xylan esterases and pectin acetyl esterases suggests that the polyaromatic fraction of suberin, like lignin, another polyaromatic structure, is linked to cell wall polysaccharides by ester bonds [22].

### Identification of extracellular proteins possibly involved in suberin degradation

The main purpose of this work was to identify extracellular proteins produced in the presence of suberin, the main constituent of potato periderm. Suberin is an insoluble lipidic biopolymer [10] and the mechanisms responsible for its degradation are poorly understood [11]. Nevertheless, some authors have suggested that actinobacteria, including *S. scabiei*[6,12], might be involved in the degradation of the aliphatic portion of suberin.

Interestingly, most proteins of the lipid metabolism class have been detected only in the supernatant of CSM (C9YTK3, C9YYE5, C9YY49, C9ZD66, C9ZGV4, C9Z6Y2, C9Z7Q3 and C9Z776) or were more abundant in this medium (C9ZCR0, C9Z5Z2 and C9Z707). Four of these proteins, a protein from the esterase-lipase family (C9YTK3), a lipolytic enzyme (C9YYE5), a glycerophosphoryl diester phosphodiesterase (C9Z5Z2) and a putative sphingolipid ceramide N-deacylase (C9ZCR0) have a predicted extracellular localisation and could thus be directly involved in suberin degradation.

Current models for suberin structure postulate that approximately 25% of the suberin structure can be depolymerized by ester cleavage reactions [8]. The predicted function of C9YTK3 and C9YYE5 suggests that these proteins could hydrolyze esters of fatty acids. They could thus be of importance in attacking the aliphatic structure of suberin or in liberating glycerol from fatty acids. Expression of the corresponding four genes was compared in CM and CSM (Table 6). The esterase/lipase gene was only slightly overexpressed in the presence of suberin while the gene coding for the lipolytic enzyme was more than ten-fold overexpressed after 2 to 5 days of incubation in the presence of suberin. Although suberin induced a considerable increase in expression of the gene encoding the lipolytic enzyme, the corresponding protein was present at a low concentration in CSM and was detected only in a 1-day-old culture medium. Komeil *et al.* (2013) [12] identified *sub1*, a potential suberinase gene in *S. scabiei* genome. The *sub1* gene was specifically expressed in the presence of suberin but the Sub1 protein has never been detected in the *S. scabiei* secretome. Because the aliphatic constituents of suberin act as cell membrane peturbants [15], a low production of lipolytic enzymes might be required for bacterial survival.

**Table 6:** **Effect of suberin on *Streptomyces scabiei *** **gene expression**

**Gene assignation**	**Protein assignation**	**Putative function**	**Relative gene expression**^**a**^			
			**Day 2**	**Day 3**	**Day 4**	**Day 5**
SCAB_3891	C9YSS1	α-galactosidase	7.3 ± 3.5^b^	6.3 ± 3.2^b^	3.4 ± 1.0^b^	3.4 ± 1.1^b^
SCAB_6001	C9YVP7	Feruloyl esterase	29.6 ± 14.8^b^	74.0 ± 24.0^b^	78.0 ± 23.6^b^	159.3 ± 14.3^b^
SCAB_51091	C9YTK3	Esterase-lipase	1.8 ± 0.6^b^	1.9 ± 0.5^b^	0.8 ± 0.1	1.5 ± 0.4
SCAB_57301	C9Z2P6	3-oxo-5.6-dehydrosuberyl-CoA semialdehyde dehydrogenase	1.7 ± 0.6^b^	2.3 ± 1.2	1.2 ± 0.6	1.0 ± 0.3
SCAB_70541	C9YYE5	Lipolytic enzyme	3.6 ± 1.3^b^	9.4 ± 4.1^b^	6.5 ± 0.9^b^	12.9 ± 1.3^b^
SCAB_74351	C9Z5Z2	Glycerophosphoryl diester phosphodiesterase	2.0 ± 0.5^b^	3.6 ± 0.9^b^	1.2 ± 0.5	1.2 ± 0.1
SCAB_78851	C9ZCR0	Sphingolipid ceramide N-deacylase	1.2 ± 0.4	4.6 ± 1.7^b^	1.8 ± 0.2^b^	1.3 ± 0.3
SCAB_79261	C9ZE96	Feruloyl esterase	298.3 ± 184.3^b^	298.6 ± 81.0^b^	301.0 ± 73.6^b^	342.0 ± 46.3^b^

^a^Data are the mean of four replicates; ^b^Gene expression was significantly different between growth conditions.

The C9Z5Z2 protein is a putative glycerophosphoryl diester phosphodiesterase involved in metabolism of glycerol and lipids and the corresponding gene was overexpressed in the first days of growth in the presence of suberin (Table 6). Glycerol has been reported to be covalently bound to the aliphatic and aromatic fractions of potato suberin, allowing the formation of a three-dimensional crosslinked network [8]. During its interaction with potato tubers, *S. scabiei* may thus release glycerol from suberin and use this compound as a carbon source. Furthermore, suberin depolymerisation by methanolysis was shown to release a set of glycerol-derived dimeric and trimeric esters [10]. Among glycerol esters, monoacylglycerols of α,ω-diacids and of ω-hydroxyacids were found in high concentrations. The putative sphingolipid ceramide N-deacylase C9ZCR0 that is overproduced in the presence of suberin might thus remove acyl groups from monoacylglycerol present in the polymer. C9ZCR0 as well as C9YSS1, a putative α-galactosidase, are related to enzymes involved in sphingolipid degradation (based on KEGG pathway database [23]) and like suberin, sphingolipids also contain long chain fatty acids. The genes encoding C9ZCR0 and C9YSS1 were overexpressed in the presence of suberin (Table 6).

Esterases exhibit activity on a wide range of substrates [24]. As such, esterases not specifically produced in the presence of suberin, for instance C9ZG71 (esterase A) and C9Z6Y6 (cholesterol esterase), might nevertheless play a role in suberin degradation (Additional file 1: Table S1). In a previous study, esterase A was detected in *S. scabiei* suberin-containing culture medium [12]. C9Z6Y6 is a widespread cholesterol esterase belonging to the lipase/esterase family [25] and it is able to hydrolyze fatty acid esters of cholesterols. Cholesterol esterases have also been characterized in bacteria such as *Pseudomonas aeruginosa*[26], *Acinetobacter* sp. [27] and *Streptomyces* spp. [28], suggesting that these bacterial enzymes do not use cholesterol as a specific substrate.

Apart from lipid metabolism proteins, accessory proteins may also actively participate in the breakdown of suberin architecture. That is the case for two feruloyl esterases (C9ZE96 and C9YVP7) included in the general function class (Tables 1 and 2). Feruloyl esterase C9ZE96 was overproduced in the presence of suberin while C9YVP7 was only found in CSM. Both feruloyl esterase genes were clearly overexpressed in the presence of suberin (between 30 to 340 times from days 2 to 5, Table 6). The potato suberin feruloyl transferase FHT, which catalyzes the transfer of ferulic acid to ω-hydroxyfatty acids and fatty alcohols, was shown to be essential for periderm maturation [29], and potatoes deficient in FHT display a periderm that is over ten times more permeable to water compared to wild-type potatoes [30]. Since suberin structure models suggest that ferulate links the aliphatic fraction to the aromatic fractions of suberin [10], feruloyl esterases may possibly disassociate the two suberin domains, making the substrate more accessible to hydrophilic enzymes. Alternatively or concomitantly, these enzymes may, as in some fungi, be responsible for cleaving ester links between polysaccharides such as xylan or pectin and ferulic acid, an aromatic residue [31].

Bacterial degradation of suberin aromatic fractions has, to our knowledge, never been documented. Only one extracellular protein in CSM could be linked to the degradation of aromatic compounds (Table 2). C9Z2P6 is a putative 3-oxo-5,6-dehydrosuberyl-CoA semialdehyde dehydrogenase that belongs to the phenylacetate catabolic pathway of aromatic compounds [32]. The gene encoding C9Z2P6 was overexpressed approximately 2-fold in the presence of suberin after the first day of growth (Table 6).

Suberin is a determining factor in the outcome of *S. scabiei*-potato tuber interaction. Suberin induces the onset of virulence mechanisms of *S. scabiei*[5]. In potato tuber, suberin biosynthesis is induced in response of *S. scabiei* infection offering a physical protection to pathogen entry [33]. A recent study has effectively shown that enhanced suberin production in potato tubers provides protection against common scab [34]. Degradation of suberin by *S. scabiei* may contribute to nutrient acquisition during both parasitic and saprophytic modes of life. Nevertheless, elucidating the involvement of suberin in the different steps of bacterial infection is still difficult as the suberin degradation process remains highly speculative.

## Conclusions

This study has allowed the identification of various extracellular enzymes that could be involved in suberin degradation (lipolytic enzymes, deacylases, feruloyl esterases) or in degradation of other potato cell wall constituents. Cellulases, xylanases or pectinases associated with *S. scabiei* have never been characterized although their role in pathogenicity may be of importance. Presence in *S. scabiei* secretome of numerous enzymes implicated in carbohydrate metabolism is unlikely to be attributable to sugar contamination of the suberin polymer, suberin rather appears to stimulate the production of such enzymes. Further study on these proteins could provide a new source of knowledge to unravel the molecular basis of *S. scabiei* virulence mechanisms.

## Methods

### Bacteria, growth conditions and inoculation

The pathogenic *Streptomyces scabiei* strain EF-35 was isolated from a common scab lesion from a potato tuber collected in Canada [2]. Bacterial inoculum was prepared as follows. Approximately 10^8^ spores were added to 25 mL of yeast malt extract (YME, 4 g/L of glucose, 4 g/L of yeast extract and 10 g/L of malt extract; BD, Detroit, MI, USA) and incubated with shaking (250 r/min) for 48 h at 30°C. The bacterial culture was then centrifuged (2500 × *g*) for 5 min and the supernatant discarded. Bacterial pellets were subsequently resuspended in 5 volumes of 0.85% NaCl. In all experiments, an inoculum of 200 μL was transferred to 50 mL of minimal medium supplemented with 0.1% suberin and 0.05% casein hydrolysate (Sigma, St. Louis, MO, USA), or casein hydrolysate only. Suberin was extracted from potato tubers according to Lerat *et al*. (2012) [6]. Three culture replicates for each medium were incubated with shaking (250 r/min) at 30°C for 1, 3 or 5 days.

### Extracellular protein extraction

The protein concentrations of *S. scabiei* supernatant samples were measured according to Bradford (1976) [35] with bovine serum albumin used as a standard. The absorbance of the solution at 595 nm was measured after 5 min of incubation at room temperature. A standard curve prepared with known concentrations of bovine serum albumin was used to determine the sample protein concentrations.

Extracellular proteins were recovered by centrifuging bacterial cultures at 2500 × *g* for 15 min at 4°C. Proteins in the supernatants were concentrated to a final volume of 500 μL using Amicon® Ultra-15 Centrifugal Filters-3 K followed by addition of 5 volumes of 100% pre-chilled acetone. After 3 h of incubation at 20°C, proteins were recovered by centrifugation (14000 × *g*, 20 min, 4°C). Protein pellets were air dried and resuspended in 80 μL of a buffer composed of 8 M urea, 2% (w/v) CHAPS, 2% (v/v) IPG buffer pH 4**–**7 (GE Healthcare, Buckinghamshire, UK), 18.15 mM DTT and 0.002% bromophenol blue stock solution in 50 mM Tris-base. A centrifugation (14000 × *g*) was then carried out for 5 min at 4°C to remove insoluble material.

### Enzymatic assays

Cellulase, licheninase and xylanase activities in *S. scabiei* culture supernatants were determined according to Lever (1972) [36]. Briefly, each supernatant sample (100 μL) was added to 400 μL of 0.1% (w/v) of carboxymethylcellulose (CMC) or 0.1% (w/v) xylan or 0.1% (w/v) lichenin and the mixtures were incubated at 50°C for 30 min. The enzymatic reaction was stopped by adding 1 mL of PAHBAH solution (NaOH 5 M, trisodium citrate 0.5 M, NaSO_3_ 1 M, CaCl_2_ 0.2 M and 10 g/L of p-hydroxy benzoic acid hydrazide). Samples were subsequently boiled for 30 min to allow color development. The vials were then placed on ice for 5 min. Insoluble material was eliminated by centrifugation (14000 × *g*, 5 min). The same procedure was carried out for the blank control samples, but PAHBAH solution was added to the supernatant sample before incubation at 50°C. The optical density of each test and blank samples was determined at 405 nm with a spectrophotometer (Ultrospec 3000-Biochrom). One unit of enzyme activity was defined as the amount of enzyme releasing 1 μmol of reducing sugar per min.

### One-dimensional gel electrophoresis

Extracellular proteins were subjected to sodium dodecyl sulfate-polyacrylamide gel electrophoresis (10% SDS-PAGE). Each protein sample consisted of 9 μL of concentrated proteins and 3 μL of loading buffer (0.5 M Tris–HCl, pH 6.8, 50% [v/v] glycerol, 10% [w/v] SDS, 5% [v/v] β-mercaptoethanol, and 0.05% [w/v] bromophenol blue) in a 12 μL final volume. The proteins were denaturated by incubating the samples at 100°C for 5 min before electrophoresis. Electrophoresis was carried out using a BioRad Mini Protean® Tetra Cell (Bio-Rad, Hercules, CA, USA) at 100 V for 60 min with a 3-(N-morpholino) propanesulfonic acid (MOPS) running buffer containing 50 mM MOPS, 50 mM Tris, 0.1% SDS, and 0.03% (w/v) EDTA. The protein molecular weight markers used were PageRuler™ Prestained Protein Ladder (Thermo Scientific, Ottawa, Canada). Proteins were stained with Coomassie brilliant blue R-250 (Bio-Rad) [2]. Individual protein bands were excised from the SDS-PAGE gels and separated into two groups according to protein band intensity (low and high intensity).

### In-gel digestion of proteins and mass spectrometry

In-gel digestion and mass spectrometry were carried out at the Proteomics Platform of the Quebec Genomics Center (Quebec City, Canada). Proteins were in-gel digested with trypsin using a MassPrep liquid handling robot (Waters, Milford, MA, USA) according to Shevchenko *et al.* (1996) [37] with modifications as suggested by Havliš *et al.* (2003) [38]. Briefly, the excised slices were destained in a solution containing 50 μL of 50 mM ammonium bicarbonate and 50 μL acetonitrile, washed once with 50 μL of 100 mM ammonium bicarbonate and dehydrated with 50 μL of acetonitrile. The proteins were in-gel reduced with 10 mM DTT for 30 min at 37°C and alkylated with 55 mM iodoacetamide for 30 min at room temperature. Proteins were digested with 105 mM sequencing grade modified porcine trypsin (Promega, Madison, WI, USA) at 58°C for 1 h. Digestion products were first extracted with a solution of 1% formic acid and 2% acetonitrile, then with a solution of 1% formic acid and 50% acetonitrile. The recovered peptide extracts were pooled, dried in a vacuum centrifuge and resuspended in 5 μL of 0.1% formic acid. Peptides were separated in a PicoFrit column BioBasic C18, 10 cm × 0.075 mm internal diameter (New Objective, Woburn, MA, USA) with a linear gradient (2% to 50% acetonitrile containing 0.1% formic acid) in 30 min at 200 nl/min. The samples were then transferred on a Thermo Surveyor MS pump connected to a LTQ linear ion trap mass spectrometer (Thermo Electron, San Jose, CA, USA) equipped with a nanoelectrospray ion source (Thermo Electron). Xcalibure 2.0 software was used for mass spectra acquisition. Each full-scan mass spectrum (400–2000 m/z) was followed by collision-induced dissociation of the seven most intense ions (30 s dynamic exclusion duration and 35% relative collisional fragmentation energy).

### Interpretation of tandem MS spectra

All MS/MS spectra were analysed for peptide identification using Mascot (Matrix Science, London, UK; version 2.2.0). Mascot parameters were set to search the *Streptomyces* Uniref100 database, based on trypsin digestion, with a fragment ion mass tolerance of 0.5 Da and a parent ion tolerance of 2.0 Da. The following search criteria were used: two missed cleavages were allowed, iodoacetamide derivative of cysteine was specified as a fixed modification and oxidation of methionine was specified as a variable modification. Peptide tolerance was 2.0 Da for the precursor and 0.5 Da for MS/MS. Score Mascot corresponded to 10 × log(P), where P is the probability that the observed match with a given MS/MS spectra is a random event.

### Protein label-free spectral counting, identification and characterisation

Scaffold software program (version Scaffold 4.0.5, Proteome Software, Portland, OR, USA) was used to group peptides into protein and sum spectral counts for each protein. Protein identifications were accepted if they obtained a 99% minimum protein ID probability and presented a minimum of two unique peptides in which the cut offs for peptide thresholds were 90%. Identified proteins were re-annotated and queried against GenBank sequence databases. Protein functions and assignment to functional groups were predicted using tools such as PRIAM [39], CAZy database [19], KEGG resources [40], COG database [41] and MicrobesOnLine resources [42]. Cellular localization of the proteins was predicted by Phobius [43], SignalP 4.1 [44], SecretomeP [45], TatP [46] and Tatfind 1.4 [47] analysis.

### Analysis of relative gene expression

The expression of genes SCAB_6001, SCAB_51091, SCAB_70541, SCAB_74351, SCAB_78851, SCAB_79251 and SCAB_84861 was monitored over time. *S. scabiei* EF-35 was grown for 5 days in casein-containing minimal medium, supplemented or not with suberin (see above for details, four replicates per medium). From 2 to 5 days after inoculation, bacterial cultures were sub-sampled (10 mL) every 24 h to extract total RNA. Sampling procedures, RNA extraction and cDNA synthesis were carried out as in Lerat *et al*. (2010) [5]. Real-time RT-PCR was then performed on 2 μL of 10× diluted cDNA (in a final volume of 20 μL) using iTaq Universal SYBR Green Supermix (Bio-Rad). Primers used for the amplification of the seven above-mentioned genes and the reference gene *gyrA* are supplied in supplementary data (Additional file 3: Table S3). PCR conditions were: 3 min at 95°C followed by 35 cycles of 15 s at 95°C and 30 s at 60°C. Relative gene expression was calculated according to Pfaffl (2001) [48].

## Abbreviations

LC-MS/MS: Liquid chromatography-mass spectrometry/mass spectrometry; MW: Molecular weight; CM: Casein medium; CSM: Casein suberin medium.

## Competing interests

The authors declare that they have no competing interests.

## Authors’ contributions

DK and RPR performed proteomics experiments and enzymatic assays. SL performed real-time RT-PCR experiments. AMSB analyzed proteomics data. CB supervised the project. All authors participated to the manuscript writing. All authors read and approved the final manuscript.

## Supplementary Material

Additional file 1: Table S1 Proteins produced by *Streptomyces scabiei* found in both casein-suberin and casein media. Click here for file

Additional file 2: Table S2 Proteins specifically produced by *Streptomyces scabiei* in casein medium. Click here for file

Additional file 3: Table S3 Primers used in this study for real-time RT-PCR. Click here for file
