# Supplementary material for: Comparative secretome analysis of Streptomyces scabiei during growth in the presence or absence of potato suberin
Source: Proteome Sci. 2014 Jun 25;12:35. doi: 10.1186/1477-5956-12-35 (PMC4098958; doi:10.1186/1477-5956-12-35)
Supplement: Additional file 1: Table S1 — Proteins produced by Streptomyces scabiei found in both casein-suberin and casein media. [file 1477-5956-12-35-S1.pdf]

Additional file 1: Table S1. Proteins produced by *Streptomyces scabiei* found in both casein-suberin and casein media.

Protein assignation[a]	Gene assignation	Putative function	Abundance (spectrum count/MW[b]) in casein-suberin medium			Abundance (spectrum count/MW[b]) in casein medium		
			**Day 1**	**Day 3**	**Day 5**	**Day 1**	**Day 3**	**Day 5**
C9ZBE2	SCAB_0601	Secreted protein	nd[c]	0.09 ± 0.05	0.06 ± 0.03	0.01 ± 0.01	0.03 ± 0.03	0.02 ± 0.01
C9ZBE8	SCAB_0651	Secreted protein	nd	0.02 ± 0.03	0.03 ± 0.04	nd	0.05 ± 0.05	0.04 ± 0.05
C9ZEJ5	SCAB_2381	Secreted protein	0.11 ± 0.00	0.04 ± 0.06	nd	0.04 ± 0.06	nd	nd
C9ZG64	SCAB_2941	Secreted protein	0.19 ± 0.16	0.76 ± 0.18	0.55 ± 0.17	0.13 ± 0.07	nd	nd
C9ZG71	SCAB_3021	Esterase A	0.14 ± 0.20	0.58 ± 0.31	0.23 ± 0.12	0.23 ± 0.10	0.17 ± 0.19	0.21 ± 0.17
C9ZG76	SCAB_3071[d]	Serine/threonine protein kinase	0.08 ± 0.08	0.22 ± 0.08	0.11 ± 0.05	0.01 ± 0.02	nd	nd
C9ZG88	SCAB_3201	Secreted protein	0.08 ± 0.07	nd	nd	0.03 ± 0.05	nd	nd
C9YUA5	SCAB_4761	Secreted protein	0.33 ± 0.23	2.47 ± 1.41	1.18 ± 0.42	0.25 ± 0.10	0.07 ± 0.08	0.02 ± 0.03
C9YUC2	SCAB_4931	Secreted protein	0.01 ± 0.02	0.03 ± 0.03	0.02 ± 0.04	0.03 ± 0.03	nd	0.01 ± 0.01
C9YUG2	SCAB_5351	ABC-type sugar transport system protein	0.19 ± 0.10	0.37 ± 0.05	0.29 ± 0.08	0.12 ± 0.03	0.37 ± 0.04	0.45 ± 0.15
C9YVL6	SCAB_5671	Secreted protein	0.07 ± 0.07	0.37 ± 0.14	0.10 ± 0.12	0.23 ± 0.11	0.26 ± 0.24	0.22 ± 0.22
C9YX78	SCAB_6661	Exported protein	0.20 ± 0.13	0.42 ± 0.08	0.33 ± 0.13	0.29 ± 0.08	0.01 ± 0.03	nd
C9YXA8	SCAB_6971	Glycine betaine-binding Lipoprotein, ABC-type transport	nd	0.24 ± 0.22	0.08 ± 0.12	nd	0.12 ± 0.10	0.30 ± 0.24
C9YYN8	SCAB_7551	Secreted protein	0.16 ± 0.04	0.76 ± 0.10	0.69 ± 0.30	0.40 ± 0.07	0.39 ± 0.15	0.28 ± 0.18
C9YYQ6	SCAB_7741	Membrane protein	0.20 ± 0.05	0.26 ± 0.07	0.19 ± 0.07	0.13 ± 0.01	0.02 ± 0.02	nd
C9Z074	SCAB_8221	Uncharacterized protein	0.00 ± 0.01	0.07 ± 0.07	0.15 ± 0.11	0.23 ± 0.25	0.01 ± 0.01	0.01 ± 0.02
C9Z082	SCAB_8301	Secreted protease	0.07 ± 0.07	0.04 ± 0.01	nd	0.02 ± 0.02	nd	nd
C9Z0C3	SCAB_8741	Solute-binding lipoprotein	0.08 ± 0.00	0.34 ± 0.12	0.11 ± 0.04	nd	0.07 ± 0.05	0.07 ± 0.06
C9Z0C9	SCAB_8801	Subtilase-type protease inhibitor	2.20 ± 0.52	4.00 ± 0.97	3.76 ± 0.67	0.62 ± 0.30	0.06 ± 0.09	nd
C9Z3C3	SCAB_10031	Secreted protein	0.02 ± 0.04	0.10 ± 0.04	0.02 ± 0.04	0.04 ± 0.07	nd	nd

C9Z516	SCAB_11521	Lipoprotein	0.99 ± 0.19	1.29 ± 0.33	0.89 ± 0.18	0.20 ± 0.06	nd	nd
C9Z6J9	SCAB_12241	Secreted protein	0.03 ± 0.06	0.35 ± 0.30	0.03 ± 0.03	0.05 ± 0.05	nd	nd
C9Z6Q3	SCAB_12851	Secreted peptidase	0.17 ± 0.08	0.44 ± 0.21	0.14 ± 0.09	0.01 ± 0.02	nd	nd
C9ZD42	SCAB_16351	Secreted protein	0.02 ± 0.03	0.34 ± 0.14	0.08 ± 0.03	0.02 ± 0.03	0.01 ± 0.02	0.01 ± 0.02
C9ZD55	SCAB_16481	Membrane protein	0.16 ± 0.15	0.46 ± 0.15	0.26 ± 0.08	0.20 ± 0.07	0.27 ± 0.06	0.17 ± 0.07
C9ZGH2	SCAB_18141[d]	Secreted protein	0.14 ± 0.07	0.44 ± 0.06	0.20 ± 0.09	0.27 ± 0.12	0.27 ± 0.01	0.25 ± 0.07
C9YT45	SCAB_19371[d]	Cellulose-binding protein	0.06 ± 0.04	0.09 ± 0.02	0.07 ± 0.02	0.04 ± 0.00	nd	nd
C9YT47	SCAB_19401	Secreted protein	nd	0.12 ± 0.15	0.10 ± 0.04	0.05 ± 0.08	nd	nd
C9YT56	SCAB_19491	Exported protein	0.06 ± 0.10	0.12 ± 0.03	0.02 ± 0.04	0.14 ± 0.05	nd	nd
C9YT70	SCAB_19641	Uncharacterized protein	0.02 ± 0.04	nd	nd	0.02 ± 0.02	nd	nd
C9YUJ5	SCAB_19751	Secreted protein	0.77 ± 0.31	0.28 ± 0.04	0.06 ± 0.04	0.07 ± 0.08	nd	nd
C9YUK3	SCAB_19841	Aliphatic sulfonate ABC transporter substrate-binding protein	0.32 ± 0.14	0.49 ± 0.14	0.33 ± 0.12	0.20 ± 0.06	0.79 ± 0.27	0.80 ± 0.33
C9YVX8	SCAB_21021	Xylose ABC transporter substrate-binding protein	0.12 ± 0.13	0.84 ± 0.27	0.63 ± 0.12	0.06 ± 0.04	0.05 ± 0.07	0.10 ± 0.07
C9YVY6	SCAB_21101[d]	Aconitate hydratase	0.05 ± 0.04	nd	nd	0.02 ± 0.02	nd	0.01 ± 0.02
C9YYV4	SCAB_22951	Acetyl-xylan esterase	0.04 ± 0.04	0.43 ± 0.19	0.25 ± 0.16	0.11 ± 0.07	0.20 ± 0.16	0.17 ± 0.15
C9Z0L9	SCAB_24621	Uncharacterized protein	0.07 ± 0.08	0.23 ± 0.08	0.09 ± 0.08	0.21 ± 0.00	0.36 ± 0.09	0.34 ± 0.15
C9Z204	SCAB_24891	Glutamate uptake system binding subunit	0.13 ± 0.12	0.89 ± 0.31	0.74 ± 0.13	0.40 ± 0.16	1.26 ± 0.21	1.20 ± 0.46
C9Z3S4	SCAB_26361	Membrane-anchored protein	0.19 ± 0.17	0.33 ± 0.10	0.26 ± 0.08	0.11 ± 0.07	nd	nd
C9Z578	SCAB_26841	Serine protease	nd	0.38 ± 0.18	0.14 ± 0.09	0.19 ± 0.10	0.22 ± 0.24	0.14 ± 0.11
C9Z5D4	SCAB_27411	Oligopeptide-binding transport system protein	0.01 ± 0.02	0.27 ± 0.22	0.25 ± 0.08	nd	0.14 ± 0.16	0.07 ± 0.09
C9Z6U2	SCAB_27811	Uncharacterized protein	0.50 ± 0.19	0.05 ± 0.02	0.01 ± 0.01	0.01 ± 0.01	nd	nd
C9Z6Y6	SCAB_28271	Cholesterol estérase	0.29 ± 0.13	0.68 ± 0.22	0.35 ± 0.17	0.72 ± 0.22	0.93 ± 0.40	0.69 ± 0.29
C9Z8E5	SCAB_28731	Chitinase C	0.04 ± 0.01	0.30 ± 0.11	0.27 ± 0.16	0.06 ± 0.02	0.29 ± 0.08	0.20 ± 0.08

C9Z8G4	SCAB_28921	secreted serine-type Endopeptidase	0.01 ± 0.01	nd	nd	nd	0.06 ± 0.06	0.09 ± 0.08
C9Z8L7	SCAB_29411	Membrane protein	0.02 ± 0.03	nd	nd	nd	0.07 ± 0.09	0.04 ± 0.04
C9ZAD0	SCAB_30191	Extracellular sugar-binding protein	nd	0.19 ± 0.13	0.10 ± 0.13	nd	0.01 ± 0.01	nd
C9ZAF2	SCAB_30421[d]	3-phosphoshikimate 1-carboxyvinyltransferase	0.01 ± 0.01	nd	nd	nd	0.01 ± 0.03	0.01 ± 0.02
C9ZBV7	SCAB_31131	Protease	0.06 ± 0.05	0.05 ± 0.01	0.03 ± 0.02	0.01 ± 0.01	nd	nd
C9ZD97	SCAB_31531	BldKB-like transport system extracellular solute-binding protein	0.01 ± 0.01	nd	nd	0.01 ± 0.02	nd	0.05 ± 0.04
C9ZF82	SCAB_33691	Transglycosylase domain-containing protein	0.51 ± 0.15	1.06 ± 0.57	0.81 ± 0.28	0.09 ± 0.04	nd	nd
C9ZGN8	SCAB_33851	Chinitase A	0.07 ± 0.06	0.13 ± 0.06	0.12 ± 0.03	0.03 ± 0.01	0.05 ± 0.05	0.04 ± 0.02
C9ZGS4	SCAB_34211	Uncharacterized protein	0.15 ± 0.07	0.22 ± 0.05	0.06 ± 0.02	0.16 ± 0.05	0.08 ± 0.10	0.09 ± 0.09
C9ZGU9	SCAB_34471	Lipoprotein	0.37 ± 0.18	0.22 ± 0.09	0.02 ± 0.02	0.03 ± 0.03	nd	nd
C9YT92	SCAB_34981	Lipoprotein	0.05 ± 0.04	0.68 ± 0.47	0.50 ± 0.36	0.14 ± 0.05	0.86 ± 0.27	0.81 ± 0.50
C9YTC3	SCAB_35291[d]	Succinate dehydrogenase flavoprotein subunit	0.01 ± 0.02	0.03 ± 0.03	0.02 ± 0.02	0.01 ± 0.01	0.01 ± 0.01	nd
C9YTD0	SCAB_35361	Secreted protein	0.83 ± 0.52	0.94 ± 0.29	0.38 ± 0.10	0.46 ± 0.16	1.30 ± 0.44	0.86 ± 0.24
C9YTG2	SCAB_35681[d]	Malate dehydrogenase	0.22 ± 0.19	0.13 ± 0.12	0.03 ± 0.03	0.30 ± 0.11	0.04 ± 0.03	0.01 ± 0.01
C9YUX5	SCAB_36201[d]	IMP dehydrogenase/ GMP reductase (RefSeq)	0.02 ± 0.03	0.01 ± 0.01	0.01 ± 0.01	0.07 ± 0.03	nd	nd
C9YW51	SCAB_36671[d]	DNA-directed RNA polymerase subunit alpha	0.02 ± 0.03	nd	nd	0.01 ± 0.02	nd	nd
C9YW53	SCAB_36691[d]	30S ribosomal protein S13	0.05 ± 0.08	nd	nd	0.10 ± 0.04	nd	nd
C9YW92	SCAB_37091[d]	Elongation factor Tu 1	0.25 ± 0.22	0.03 ± 0.03	nd	0.02 ± 0.04	nd	nd
C9YWB0	SCAB_37271[d]	Aspartate aminotransferase	0.11 ± 0.10	0.02 ± 0.03	nd	0.03 ± 0.05	nd	nd
C9YWC0	SCAB_37371[d]	Hypothetical protein	0.02 ± 0.03	0.02 ± 0.03	nd	0.04 ± 0.06	nd	nd

(+1)								
C9YXR8	SCAB_37611	Aminopeptidase	0.18 ± 0.08	0.71 ± 0.18	0.55 ± 0.27	0.71 ± 0.21	1.26 ± 0.53	1.12 ± 0.72
C9YXT6	SCAB_37811	Membrane protein	0.38 ± 0.11	0.44 ± 0.05	0.11 ± 0.08	0.01 ± 0.01	nd	nd
C9YXX5	SCAB_38231	Cold shock protein	nd	0.48 ± 0.30	0.38 ± 0.08	0.14 ± 0.14	0.02 ± 0.06	nd
C9YZA8	SCAB_38901	Membrane protein	0.35 ± 0.06	1.41 ± 0.12	0.89 ± 0.26	0.10 ± 0.07	nd	0.03 ± 0.03
C9YZB9	SCAB_39021	Uncharacterized protein	0.03 ± 0.04	0.21 ± 0.06	0.15 ± 0.08	0.09 ± 0.08	nd	nd
C9YZD0	SCAB_39131	Membrane protein	0.32 ± 0.13	0.68 ± 0.05	0.51 ± 0.21	0.01 ± 0.01	nd	nd
C9Z0Q1	SCAB_39341[d]	Uncharacterized protein	0.07 ± 0.05	0.09 ± 0.05	0.03 ± 0.02	0.04 ± 0.02	nd	nd
C9Z0X0	SCAB_40041	Lipoprotein	0.24 ± 0.14	0.70 ± 0.12	0.69 ± 0.08	0.41 ± 0.08	0.63 ± 0.19	0.24 ± 0.17
C9Z5G9	SCAB_42541[d]	Chaperone protein DnaK 2	0.08 ± 0.08	0.04 ± 0.07	nd	0.09 ± 0.06	0.08 ± 0.05	nd
C9Z5M7	SCAB_43121	Uncharacterized protein	0.04 ± 0.02	0.13 ± 0.05	0.09 ± 0.03	0.03 ± 0.04	0.02 ± 0.04	0.01 ± 0.02
C9Z722	SCAB_43501	Carboxypeptidase	0.31 ± 0.11	0.24 ± 0.11	0.15 ± 0.06	0.51 ± 0.05	0.15 ± 0.15	0.07 ± 0.08
C9Z759	SCAB_43891	Secreted protein	0.16 ± 0.09	0.43 ± 0.09	0.43 ± 0.24	0.37 ± 0.09	0.06 ± 0.06	0.02 ± 0.05
C9Z760	SCAB_43901	Secreted hydrolase	0.79 ± 0.34	0.74 ± 0.25	0.48 ± 0.11	1.25 ± 0.04	1.66 ± 0.53	1.38 ± 0.57
C9Z765	SCAB_43951	Uncharacterized protein	0.13 ± 0.19	0.42 ± 0.15	0.23 ± 0.08	0.13 ± 0.10	0.13 ± 0.18	0.03 ± 0.06
C9Z770	SCAB_44001	Secreted protein	0.12 ± 0.16	0.04 ± 0.02	0.02 ± 0.03	nd	0.06 ± 0.07	0.01 ± 0.01
C9Z8N7	SCAB_44501	Membrane protein	nd	0.24 ± 0.19	0.03 ± 0.03	0.08 ± 0.07	nd	nd
C9Z8P9	SCAB_44621	Secreted protein	0.11 ± 0.07	0.51 ± 0.07	0.32 ± 0.15	0.22 ± 0.15	0.04 ± 0.05	nd
C9Z8Q6	SCAB_44691	Secreted protein	0.22 ± 0.07	0.12 ± 0.07	0.01 ± 0.02	0.02 ± 0.04	nd	nd
C9ZAH6 (+4)	SCAB_45481	Secreted protein	0.05 ± 0.05	0.23 ± 0.08	0.18 ± 0.08	0.04 ± 0.06	0.01 ± 0.02	nd
C9ZAJ2	SCAB_45651[d]	Peptidyl-prolyl cis-trans isomerase	0.16 ± 0.19	0.33 ± 0.21	0.19 ± 0.13	0.54 ± 0.11	0.03 ± 0.04	0.01 ± 0.02
C9ZAL8 (+15)	SCAB_45931[d]	Thioredoxin reductase	0.04 ± 0.04	0.02 ± 0.03	0.03 ± 0.03	0.09 ± 0.06	0.01 ± 0.02	0.01 ± 0.02
C9ZAM8	SCAB_46031	Secreted transglycosylase	0.17 ± 0.07	0.02 ± 0.01	nd	0.01 ± 0.01	nd	nd
C9ZC23	SCAB_46591	Copper-binding protein	nd	0.06 ± 0.01	0.02 ± 0.02	0.00 ± 0.01	0.01 ± 0.02	0.00 ± 0.01
C9ZC25	SCAB_46611	Secreted protein	0.25 ± 0.13	1.14 ± 0.44	0.79 ± 0.31	0.01 ± 0.02	nd	nd

C9ZFG9	SCAB_49021	Peptidase	0.16 ± 0.02	0.38 ± 0.06	0.03 ± 0.03	0.01 ± 0.02	nd	nd
C9ZFJ5	SCAB_49311	High-affinity phosphate-binding protein	1.15 ± 0.38	2.21 ± 0.45	1.86 ± 0.50	0.75 ± 0.16	1.05 ± 0.36	0.97 ± 0.45
C9ZH11	SCAB_49881	Secreted protein	nd	0.10 ± 0.05	0.04 ± 0.01	0.01 ± 0.01	nd	nd
C9ZH47	SCAB_50261[d]	TerD-like stress protein	0.27 ± 0.14	0.65 ± 0.22	0.52 ± 0.06	0.43 ± 0.12	0.63 ± 0.23	0.59 ± 0.20
C9YV41	SCAB_52001	Secreted protein	1.13 ± 0.57	1.63 ± 0.32	1.27 ± 0.52	0.69 ± 0.14	0.90 ± 0.19	0.76 ± 0.33
C9YV96	SCAB_52561[d]	Membrane protein	0.15 ± 0.05	0.07 ± 0.08	nd	0.03 ± 0.05	nd	nd
C9YWE9	SCAB_53001	Lipoprotein	0.46 ± 0.20	0.11 ± 0.05	0.09 ± 0.04	0.02 ± 0.03	nd	nd
C9YWG0	SCAB_53111	Secreted penicillin acylase	0.04 ± 0.04	0.02 ± 0.02	nd	0.01 ± 0.01	0.00 ± 0.01	nd
C9YXZ5	SCAB_53981	Secreted peptidase	nd	0.03 ± 0.02	0.02 ± 0.02	0.04 ± 0.02	0.06 ± 0.03	0.07 ± 0.03
C9YY35	SCAB_54421	Transglycosylase domain-containing protein	0.54 ± 0.19	0.32 ± 0.09	0.15 ± 0.13	0.06 ± 0.03	nd	nd
C9YY36	SCAB_54431	Cell wall catabolism protein	0.22 ± 0.10	0.29 ± 0.08	0.24 ± 0.09	0.03 ± 0.05	nd	nd
C9YY37	SCAB_54441[d]	Enolase	0.04 ± 0.06	nd	0.01 ± 0.01	nd	0.01 ± 0.02	0.03 ± 0.03
C9YZE4	SCAB_54701[d]	Urocanate hydratase	0.01 ± 0.02	0.06 ± 0.06	0.10 ± 0.02	0.01 ± 0.01	0.05 ± 0.03	0.00 ± 0.01
C9Z0Z3	SCAB_55741[d]	Carboxy-terminal processing protease	0.22 ± 0.06	0.08 ± 0.04	nd	0.02 ± 0.03	nd	nd
C9Z2Q2	SCAB_57371	Secreted chitin binding protein	nd	0.39 ± 0.23	0.38 ± 0.13	0.05 ± 0.05	0.41 ± 0.12	0.28 ± 0.16
C9Z473	SCAB_57981	Protein DesF, iron transport system	0.13 ± 0.12	0.19 ± 0.16	0.06 ± 0.06	0.05 ± 0.03	0.16 ± 0.08	0.10 ± 0.09
C9Z487	SCAB_58141	Integrin-like protein	0.10 ± 0.09	nd	nd	0.04 ± 0.04	nd	nd
C9Z5T6	SCAB_58971	Chorismate mutase	0.01 ± 0.03	0.01 ± 0.03	nd	0.04 ± 0.04	nd	nd
C9Z5W7	SCAB_59301	Sortase-sorted protein	0.09 ± 0.09	0.08 ± 0.02	0.01 ± 0.02	nd	0.00 ± 0.01	nd
C9Z7C3	SCAB_59681	Secreted peptidase	nd	0.01 ± 0.01	nd	nd	0.01 ± 0.02	0.03 ± 0.02
C9Z7C8	SCAB_59731[d]	Superoxide dismutase	0.08 ± 0.11	0.17 ± 0.11	0.15 ± 0.10	0.01 ± 0.02	0.20 ± 0.20	0.22 ± 0.16
C9Z7D6	SCAB_59811	Membrane protein	nd	0.12 ± 0.12	nd	nd	0.01 ± 0.02	nd
C9ZAQ3	SCAB_61791	Uncharacterized protein	0.11 ± 0.09	nd	nd	0.02 ± 0.02	nd	nd
C9ZAT6	SCAB_62141[d]	Pyruvate phosphate dikinase	0.01 ± 0.01	0.01 ± 0.01	nd	0.00 ± 0.01	nd	nd

(+22)								
C9ZDY2	SCAB_64081	Secreted protein	0.66 ± 0.41	1.77 ± 0.40	1.13 ± 0.15	0.91 ± 0.18	0.74 ± 0.36	0.79 ± 0.20
C9ZE07	SCAB_64331[d]	TerD-like stress protein	0.17 ± 0.15	0.17 ± 0.12	0.18 ± 0.06	0.33 ± 0.08	0.30 ± 0.17	0.33 ± 0.16
C9ZE08	SCAB_64341[d]	TerD-like stress protein	0.10 ± 0.10	0.15 ± 0.05	0.20 ± 0.00	0.30 ± 0.00	0.43 ± 0.07	0.58 ± 0.24
C9ZE18	SCAB_64451	Hypothetical protein	0.40 ± 0.32	0.08 ± 0.04	0.03 ± 0.04	0.07 ± 0.04	0.00 ± 0.01	nd
C9ZFN9	SCAB_65271[d]	Uncharacterized protein	0.12 ± 0.21	0.21 ± 0.14	0.02 ± 0.04	0.24 ± 0.04	0.02 ± 0.06	nd
C9ZHF3	SCAB_66761	Secreted protein	1.10 ± 0.04	0.42 ± 0.11	0.42 ± 0.10	0.26 ± 0.07	0.07 ± 0.03	0.05 ± 0.03
C9YTR7	SCAB_67061[d]	Dihydrolipoyl dehydrogenase	0.46 ± 0.43	0.59 ± 0.37	0.95 ± 0.15	0.54 ± 0.10	1.37 ± 0.43	1.63 ± 0.12
C9YTV5	SCAB_67441	Secreted protein	0.09 ± 0.06	0.22 ± 0.11	0.11 ± 0.06	0.12 ± 0.11	0.01 ± 0.03	0.00 ± 0.01
C9YTX5	SCAB_67651	Secreted protein	0.05 ± 0.05	0.02 ± 0.03	nd	0.02 ± 0.02	nd	nd
C9YWP0	SCAB_68931	Branched-chain amino acid ABC transporter substrate-binding protein	0.55 ± 0.23	1.95 ± 0.47	1.13 ± 0.15	0.67 ± 0.19	1.10 ± 0.39	1.02 ± 0.21
C9YWP7	SCAB_69011	Lytic transglycosylase	0.20 ± 0.13	0.20 ± 0.09	0.13 ± 0.10	0.13 ± 0.02	0.04 ± 0.05	0.02 ± 0.02
C9YWU0	SCAB_69441[d]	Stress-induced protein	0.02 ± 0.03	nd	nd	0.02 ± 0.03	nd	nd
C9YY62 (+5)	SCAB_69701[d]	Glyceraldehyde-3-phosphate dehydrogenase	0.01 ± 0.02	nd	nd	nd	0.07 ± 0.06	0.11 ± 0.06
C9YZU4	SCAB_71211	ssp-like secreted protease	0.09 ± 0.02	0.04 ± 0.04	nd	0.16 ± 0.06	nd	nd
C9Z2V8	SCAB_72781	Penicillin acylase	0.04 ± 0.06	0.39 ± 0.15	0.26 ± 0.07	0.13 ± 0.05	0.23 ± 0.07	0.20 ± 0.11
C9Z4D5	SCAB_73521	Peptidyl-prolyl cis-trans isomerase	0.02 ± 0.03	0.11 ± 0.09	0.05 ± 0.06	0.04 ± 0.04	0.01 ± 0.02	nd
C9Z4K8	SCAB_74261	Secreted protein	0.46 ± 0.05	0.78 ± 0.14	0.64 ± 0.07	0.02 ± 0.03	nd	nd
C9Z7Q2	SCAB_75761	Chitinase/cellulase	0.01 ± 0.01	0.03 ± 0.01	0.02 ± 0.03	0.01 ± 0.01	0.08 ± 0.06	0.06 ± 0.04
C9Z9C8	SCAB_76271[d]	Uncharacterized protein	0.02 ± 0.03	0.72 ± 0.63	0.95 ± 0.35	0.19 ± 0.15	0.11 ± 0.06	0.03 ± 0.04
C9Z9G7	SCAB_76661	Lipoprotein	0.16 ± 0.09	0.66 ± 0.19	0.33 ± 0.15	0.12 ± 0.08	0.02 ± 0.05	nd
C9ZEA9	SCAB_79401	Xylanase A	nd	0.10 ± 0.05	0.05 ± 0.05	nd	0.05 ± 0.06	0.04 ± 0.05
C9ZEC5	SCAB_79561	Secreted glycosyl hydrolase	nd	0.37 ± 0.16	0.24 ± 0.11	nd	0.14 ± 0.13	0.15 ± 0.12
C9ZHS9	SCAB_81661[d]	TerD-like stress protein	0.28 ± 0.30	0.53 ± 0.15	0.57 ± 0.10	0.55 ± 0.05	0.77 ± 0.27	0.94 ± 0.44

C9YU10	SCAB_81831	Serine-threonine protein kinase	0.01 ± 0.01	0.08 ± 0.05	0.03 ± 0.04	0.01 ± 0.02	nd	nd
C9YU17	SCAB_81901	Peptide/nickel transport system substrate-binding protein	nd	0.53 ± 0.30	0.47 ± 0.16	0.05 ± 0.09	0.00 ± 0.01	nd
C9YWW7	SCAB_82691	Endonuclease/exonuclease/phosphatase family protein	nd	0.01 ± 0.02	0.08 ± 0.07	0.06 ± 0.03	nd	nd
C9YWW9	SCAB_82711	Uncharacterized protein	0.04 ± 0.01	0.10 ± 0.03	0.06 ± 0.02	0.03 ± 0.00	0.03 ± 0.01	0.03 ± 0.01
C9Z058	SCAB_84971	Uncharacterized protein	0.03 ± 0.03	0.07 ± 0.01	0.08 ± 0.02	0.06 ± 0.01	0.08 ± 0.06	0.12 ± 0.05
C9Z064	SCAB_85031	Secreted protein	0.06 ± 0.10	nd	nd	nd	0.09 ± 0.09	0.12 ± 0.07
C9Z800	SCAB_89701	Secreted subtilisin-like protease	nd	0.03 ± 0.02	0.01 ± 0.01	0.10 ± 0.03	0.02 ± 0.02	0.04 ± 0.02
C9Z813	SCAB_89831	Secreted protein	0.23 ± 0.14	0.70 ± 0.15	0.62 ± 0.15	0.11 ± 0.05	nd	nd
C9Z823	SCAB_89921	Secreted protein	0.06 ± 0.02	0.21 ± 0.05	0.14 ± 0.05	0.07 ± 0.03	nd	nd
C9Z9T4	SCAB_90811	Secreted protein	0.21 ± 0.21	1.09 ± 0.28	1.03 ± 0.37	0.33 ± 0.15	0.68 ± 0.22	0.41 ± 0.11
C9Z9U4	SCAB_90901	Secreted protein	0.08 ± 0.14	0.81 ± 0.34	0.53 ± 0.17	0.25 ± 0.08	0.40 ± 0.41	0.24 ± 0.15

[a] Uniprot accession number; [b] Data are the mean of three replicates; [c] nd: not detected; [d] Protein with intracellular localization prediction.
