# Supplementary material for: Comparative secretome analysis of Streptomyces scabiei during growth in the presence or absence of potato suberin
Source: Proteome Sci. 2014 Jun 25;12:35. doi: 10.1186/1477-5956-12-35 (PMC4098958; doi:10.1186/1477-5956-12-35)
Supplement: Additional file 2: Table S2 — Proteins specifically produced by Streptomyces scabiei in casein medium. [file 1477-5956-12-35-S2.pdf]

Additional file 2: Table S2. Proteins specifically produced by *Streptomyces scabiei* in casein medium

Protein designation[a]	Gene designation	Putative function	Abundance (spectrum count/MW[b])		
			Day 1	Day 3	Day 5
Translational, ribosomal structure and biogenesis					
C9Z3N9	SCAB_26011[d]	Elongation factor Ts	0.04 ± 0.08	nd[c]	nd
C9YW93	SCAB_37101[d]	Elongation factor G2	0.06 ± 0.07	nd	nd
Transcription					
C9Z8G3	SCAB_28911	Putative transcriptional regulator	0.04 ± 0.01	nd	nd
C9ZHT2	SCAB_81701	Putative transcriptional regulator	0.04 ± 0.06	nd	nd
Posttranslational modification, protein turnover, chaperone					
C9Z0K0	SCAB_24411	Serine protease	0.02 ± 0.04	0.35 ± 0.08	0.19 ± 0.19
C9ZBR8	SCAB_30731	Glutaredoxin-like protein	nd	0.13 ± 0.22	0.13 ± 0.13
C9YUY4	SCAB_36291[d]	Chaperonin GroEL (HSP60 family)	0.06 ± 0.07	nd	nd
C9ZC44	SCAB_46811	Protease	0.06 ± 0.02	nd	nd
C9ZDR4	SCAB_47841	Lipoprotein	0.05 ± 0.04	0.77 ± 0.30	0.24 ± 0.12
C9ZH64	SCAB_50441[d]	Chaperonin GroEL (HSP60 family)	0.23 ± 0.25	nd	nd
C9ZHH5	SCAB_66981[d]	Serine protease	nd	0.13 ± 0.08	0.01 ± 0.01
C9YVE7	SCAB_68221	DSBA oxidoreductase	0.14 ± 0.18	0.72 ± 0.18	0.09 ± 0.09
C9YVH7	SCAB_68521	DSBA-like thioredoxin domain protein	nd	0.06 ± 0.08	nd
C9Z4H4	SCAB_73921	Serine protease	0.02 ± 0.04	nd	nd
C9Z651	SCAB_74981	Peptidyl-prolyl cis-trans isomerase	0.01 ± 0.02	0.03 ± 0.03	nd
Cell wall/membrane/envelope biogenesis					
C9Z0D2	SCAB_8831[d]	Polypeptide N-acetylgalactosaminyltransferase	nd	0.05 ± 0.04	0.01 ± 0.01
C9Z3C9	SCAB_10101	Penicillin binding protein	0.02 ± 0.04	nd	nd
C9Z544	SCAB_11801	Peptidase	0.03 ± 0.04	0.09 ± 0.05	0.05 ± 0.07
C9Z0I6	SCAB_24251	Peptidase	0.37 ± 0.00	0.36 ± 0.02	0.22 ± 0.06
C9YTE7	SCAB_35531	D-alanyl-D-alanine carboxypeptidase	0.01 ± 0.02	nd	nd
C9YUU8	SCAB_35931	Glycoside hydrolase	0.05 ± 0.07	0.03 ± 0.03	nd

C9YUV0	SCAB_35951	NlpC/P60 family protein	0.11 ± 0.05	nd	nd
C9YXW3	SCAB_38101	NLP/P60 family protein	0.08 ± 0.02	0.20 ± 0.07	0.09 ± 0.07
C9Z3V9	SCAB_41401	Penicillin-binding transpeptidase/transglycosylase	0.10 ± 0.08	nd	nd
C9ZAI2	SCAB_45551	Penicillin-binding protein	0.04 ± 0.06	0.01 ± 0.02	nd
C9ZFJ2	SCAB_49281	Transglycosylase	nd	0.09 ± 0.04	0.01 ± 0.02
C9Z7G0	SCAB_60031[d]	Rod shape-determining protein	0.05 ± 0.05	nd	nd
C9ZFR2	SCAB_65501	Cell wall glycoprotein hydrolase	0.19 ± 0.09	0.02 ± 0.03	nd
C9YZN6	SCAB_70631	Penicillin binding protein	0.03 ± 0.05	nd	nd
Signal transduction mechanism					
C9ZBP9	SCAB_30521	Protein containing a PDZ domain	0.05 ± 0.07	nd	nd
C9ZAH5	SCAB_45471[d]	Forkhead-associated protein	0.03 ± 0.03	0.08 ± 0.05	0.07 ± 0.06
C9YZK1	SCAB_55281[d]	Two-component system response regulator	0.05 ± 0.09	nd	nd
Secretion					
C9YVQ1	SCAB_6041	SecDF protein-export membrane protein	0.04 ± 0.05	0.19 ± 0.08	0.07 ± 0.01
C9ZH73	SCAB_50531	Preprotein translocase subunit SecD	0.06 ± 0.10	0.33 ± 0.05	0.08 ± 0.07
C9Z645	SCAB_74911	Protein translocase subunit SecD	0.03 ± 0.00	0.30 ± 0.16	0.10 ± 0.07
Defense mechanisms and virulence					
C9YTG5	SCAB_50691	Necrosis inducing protein	nd	0.05 ± 0.04	0.04 ± 0.06
C9ZB10	SCAB_77321	Tomatinase	0.04 ± 0.04	nd	nd
C9ZAY5	SCAB_77602	Virulence factor nec1	0.11 ± 0.10	nd	nd
Differentiation					
C9Z3A4	SCAB_86841	Spore-associated protein	0.12 ± 0.04	0.14 ± 0.07	0.10 ± 0.08
Energy production and conversion					
C9ZGR4	SCAB_34111[d]	Phosphoenolpyruvate carboxykinase (GTP)	0.04 ± 0.04	nd	nd
C9YTR6	SCAB_67051[d]	Dihydrolipoyllysine-residue succinyltransferase	0.06 ± 0.08	nd	nd
C9YTU0	SCAB_67291	Heme/copper-type cytochrome/quinol oxidase subunit 2	0.13 ± 0.12	0.44 ± 0.17	0.23 ± 0.11
C9YTU6	SCAB_67351	Cytochrome c. mono- and diheme variant	0.10 ± 0.04	0.17 ± 0.07	0.06 ± 0.05
Lipid metabolism					
C9Z1G4	SCAB_72401	Lipase	0.02 ± 0.04	0.04 ± 0.06	0.05 ± 0.09
Carbohydrate metabolism					
C9ZGB6	SCAB_3481	Levanase	nd	0.01 ± 0.01	nd

C9Z5F4	SCAB_42381	Lipoprotein transport system associated	0.02 ± 0.03	0.06 ± 0.04	0.02 ± 0.03
C9Z5U5	SCAB_59071	β-mannanase	0.03 ± 0.04	nd	nd
C9ZHD5	SCAB_66581	Maltose-binding protein	0.08 ± 0.09	0.01 ± 0.01	nd
C9ZB14	SCAB_77361	Substrate-binding protein transport system associated	nd	0.08 ± 0.07	0.01 ± 0.01
Amino acid metabolism					
C9Z0B6	SCAB_8661	Protease	0.09 ± 0.02	0.06 ± 0.03	0.03 ± 0.03
C9Z6L9	SCAB_12481	Substrate-binding protein transport system associated	nd	0.09 ± 0.03	0.01 ± 0.02
C9Z0M7	SCAB_24711	V8-like Glu-specific endopeptidase	0.20 ± 0.13	0.08 ± 0.01	0.02 ± 0.03
C9ZA98	SCAB_29881	Amino acid ABC transporter solute-binding protein	0.09 ± 0.16	0.30 ± 0.18	0.01 ± 0.02
C9ZBT4	SCAB_30901	Peptidase	0.03 ± 0.04	0.04 ± 0.04	nd
C9ZBY7	SCAB_31431	Transport system peptide-binding protein	nd	0.07 ± 0.06	nd
C9YUX1	SCAB_36161[d]	Ser/Thr protein kinase	0.04 ± 0.01	0.14 ± 0.03	0.08 ± 0.03
C9YTZ8	SCAB_67881[d]	Transglutaminase-like enzyme	0.02 ± 0.04	0.12 ± 0.08	0.01 ± 0.01
C9Z1B0	SCAB_71831	Alanine dehydrogenase	0.06 ± 0.10	nd	nd
C9Z311	SCAB_73351	Oligopeptide-binding lipoprotein	0.02 ± 0.03	0.13 ± 0.08	nd
C9Z312	SCAB_73361	Substrate-binding lipoprotein	0.12 ± 0.13	0.08 ± 0.03	0.01 ± 0.01
C9Z317	SCAB_73411	Succinylglutamate desuccinylase	0.05 ± 0.05	0.08 ± 0.03	nd
C9Z9F7	SCAB_76561	Solute binding transport lipoprotein	0.09 ± 0.14	0.17 ± 0.08	0.01 ± 0.02
C9Z9K4	SCAB_89971	Glutamate uptake system binding subunit	0.03 ± 0.03	0.41 ± 0.07	0.29 ± 0.20
Coenzyme metabolism					
C9YZJ1	SCAB_55181[d]	Adenosylhomocysteinase	0.04 ± 0.05	nd	nd
Nucleotide metabolism					
C9YUT1	SCAB_35741[d]	Bifunctional purine biosynthesis protein PurH	0.01 ± 0.02	nd	nd
Inorganic ion metabolism					
C9Z1Z0	SCAB_9831	Iron transport lipoprotein	0.02 ± 0.03	nd	nd
C9ZDM4	SCAB_47441	Copper export protein	0.01 ± 0.02	0.05 ± 0.01	0.00 ± 0.01
C9ZAS1	SCAB_61981	ABC-transporter metal-binding lipoprotein	0.33 ± 0.16	0.26 ± 0.18	0.05 ± 0.04
General function prediction only					
C9Z0A5	SCAB_8551	Serine/threonine protein kinase	nd	0.02 ± 0.04	nd
C9Z521	SCAB_11571[d]	Peptidase	nd	0.25 ± 0.17	nd
C9YUX2	SCAB_36171	Serine/threonine protein kinase	0.05 ± 0.02	0.02 ± 0.02	nd

C9YZF2	SCAB_54781	Peptidase	nd	0.01 ± 0.03	nd
C9YZL5	SCAB_55431	Serine/threonine protein kinase	0.01 ± 0.01	0.07 ± 0.05	nd
C9Z2K0	SCAB_56851	SGNH_hydrolase protein	0.12 ± 0.21	nd	nd
C9Z464	SCAB_57891	Phosphoesterase	0.05 ± 0.07	0.01 ± 0.02	nd
C9YTY1	SCAB_67711[d]	Serine/threonine protein kinase	nd	0.02 ± 0.03	nd
C9Z7K6	SCAB_75261[d]	Integration host factor	nd	0.08 ± 0.14	nd
Unknown function					
C9ZEK6	SCAB_2511		0.17 ± 0.18	0.31 ± 0.04	0.14 ± 0.07
C9ZEN8	SCAB_2861		0.02 ± 0.04	0.30 ± 0.06	0.16 ± 0.08
C9YVP6	SCAB_5991		0.04 ± 0.07	0.15 ± 0.20	0.08 ± 0.07
C9Z898	SCAB_13691		0.15 ± 0.17	0.01 ± 0.02	nd
C9YVV1	SCAB_20751		0.07 ± 0.07	0.04 ± 0.00	nd
C9YVV3	SCAB_20771		nd	0.04 ± 0.04	nd
C9Z3M9	SCAB_25911		0.03 ± 0.04	0.18 ± 0.06	0.03 ± 0.04
C9Z704	SCAB_28451		0.03 ± 0.05	0.28 ± 0.05	0.31 ± 0.17
C9ZGR1	SCAB_34081		0.01 ± 0.01	0.10 ± 0.06	0.05 ± 0.01
C9YTG1	SCAB_35671		0.03 ± 0.05	0.26 ± 0.16	0.25 ± 0.11
C9YW48	SCAB_36641		0.20 ± 0.24	0.12 ± 0.02	0.06 ± 0.00
C9YW90	SCAB_37071		nd	0.03 ± 0.05	0.03 ± 0.05
C9YZA7	SCAB_38891		nd	0.04 ± 0.04	nd
C9YZB8	SCAB_39011		0.11 ± 0.06	0.20 ± 0.05	0.06 ± 0.04
C9Z0P4	SCAB_39271			0.22 ± 0.10	0.36 ± 0.05
C9Z0Q5	SCAB_39381		0.02 ± 0.04	0.10 ± 0.02	0.06 ± 0.06
C9Z3W8	SCAB_41491[d]		0.01 ± 0.03	nd	nd
C9Z5J2	SCAB_42771		0.76 ± 0.72	nd	0.02 ± 0.03
C9Z7A3	SCAB_44341		nd	0.06 ± 0.03	0.03 ± 0.05
C9Z8P6	SCAB_44591		nd	0.12 ± 0.10	0.01 ± 0.02
C9ZAI3	SCAB_45561		nd	0.07 ± 0.03	0.03 ± 0.04
C9ZAJ6	SCAB_45691		0.01 ± 0.02	0.03 ± 0.03	nd
C9ZC26	SCAB_46621		0.13 ± 0.08	0.64 ± 0.17	0.24 ± 0.08
C9ZC73	SCAB_47121		0.05 ± 0.09	0.07 ± 0.03	0.04 ± 0.03

C9ZDS2	SCAB_47931	0.06 ± 0.04	0.01 ± 0.01	nd
C9ZH28	SCAB_50061	0.13 ± 0.04	0.19 ± 0.07	0.01 ± 0.03
C9YTI5	SCAB_50911	0.07 ± 0.13	0.07 ± 0.06	0.02 ± 0.04
C9YTJ9	SCAB_51051	0.04 ± 0.04	0.08 ± 0.03	0.03 ± 0.03
C9YV42	SCAB_52011	0.13 ± 0.19	0.36 ± 0.24	0.17 ± 0.09
C9YXY9	SCAB_53921	0.05 ± 0.02	0.00 ± 0.01	nd
C9YZE8	SCAB_54741	0.02 ± 0.03	0.13 ± 0.04	0.09 ± 0.02
C9YZI3	SCAB_55101	0.03 ± 0.05	0.04 ± 0.02	0.01 ± 0.01
C9YZK3	SCAB_55301	0.06 ± 0.07	0.14 ± 0.08	0.03 ± 0.02
C9Z7B9	SCAB_59641	nd	0.15 ± 0.16	0.09 ± 0.03
C9ZE17	SCAB_64441	0.03 ± 0.04	nd	nd
C9ZFR7	SCAB_65551[d]	nd	0.13 ± 0.10	nd
C9ZHF7	SCAB_66801	0.09 ± 0.04	0.59 ± 0.31	0.32 ± 0.22
C9YTT6	SCAB_67251	0.02 ± 0.02	0.23 ± 0.12	0.10 ± 0.06
C9YYF1	SCAB_70601	0.02 ± 0.04	0.13 ± 0.03	0.01 ± 0.02
C9Z4L1	SCAB_74291	0.07 ± 0.06	0.21 ± 0.07	0.02 ± 0.04
C9ZCN2	SCAB_78561	0.05 ± 0.07	nd	nd
C9ZCQ1	SCAB_78761[d]	0.11 ± 0.15	nd	nd
C9YU74	SCAB_82491	nd	0.14 ± 0.05	0.04 ± 0.04
C9Z4V5	SCAB_87971[d]	0.05 ± 0.01	0.14 ± 0.06	0.04 ± 0.02
C9Z687	SCAB_88211	nd	0.33 ± 0.31	0.29 ± 0.13
C9Z7Y9	SCAB_89571	nd	0.08 ± 0.08	0.03 ± 0.05
C9Z801	SCAB_89711	nd	0.09 ± 0.04	0.06 ± 0.05

[a] Uniprot accession number; [b] Data are the mean of three replicates; [c] nd: not detected; [d] Protein with intracellular localisation prediction.
