# Supplementary material for: Comparative secretome analysis of Streptomyces scabiei during growth in the presence or absence of potato suberin
Source: Proteome Sci. 2014 Jun 25;12:35. doi: 10.1186/1477-5956-12-35 (PMC4098958; doi:10.1186/1477-5956-12-35)
Supplement: Additional file 3: Table S3 — Primers used in this study for real-time RT-PCR. [file 1477-5956-12-35-S3.pdf]

Additional file 3: Table S3. Primers used in this study for real-time RT-PCR.

Gene assignation	Putative function	Primers sets (5'-3')
SCAB_3891	α-galactosidase	For- CAGCAGATCGCGCAGTT<br>Rev-GTGGAAGCTGTTGGGATTGA
SCAB_6001	Feruloyl esterase	For-GCGGCAAGAACCGGAGCTTCATCCT<br>Rev-AGTGGAACCCGAACACCACCCGGTA
SCAB_51091	Esterase-lipase	For-TCCTGCCGTACATCAACACGATCA<br>Rev-AAGTAGTTGATCTTCTTCGCGCCG
SCAB_57301	3-oxo-5.6-dehydrosuberyl-CoA semialdehyde dehydrogenase	For-CGTGAACCTCGGCGTGAAGTG<br>Rev-GAGTTCAGCACGTCCTGGGAGTA
SCAB_70541	Lipolytic enzyme	For-GTTCGGGCACAACGACTCCAAG<br>Rev-CGTCCAGGTAGCGGAGCAGAT
SCAB_74351	Glycerophosphoryl diester phosphodiesterase	For-AACACCTTCCTGCCCGCGAACTTCC<br>Rev-AGTACACCTTGAACGCGCCGAACGC
SCAB_78851	Sphingolipid ceramide N-deacylase	For-TGCTCGATCTACCCGTTCAAGGAA<br>Rev-TGTCGATGTAGTCCTGAAGGTCGT
SCAB_79261	Feruloyl esterase	For-GGCAAGAGCCGCAGCTTCATCCTCA<br>Rev-CAGTGGAAGGCGAAGATCAGCCGGT
SCAB_24291	Gyrase A (*gyrA*)	For-GGACATCCAGACGCAGTACA<br>Rev-CTCGGTGTTGAGCTTCTCCT
